# *Salmonella* Effector SteA Suppresses Proinflammatory Responses of the Host by Interfering With IκB Degradation

**DOI:** 10.3389/fimmu.2019.02822

**Published:** 2019-12-10

**Authors:** Aakanksha Gulati, Rhythm Shukla, Arunika Mukhopadhaya

**Affiliations:** Department of Biological Sciences, Indian Institute of Science Education and Research Mohali, Sahibzada Ajit Singh Nagar, India

**Keywords:** *Salmonella enterica* serovar Typhimurium, SteA, proinflammatory responses, IκB degradation, E3 ligase

## Abstract

*Salmonella enterica* serovar Typhimurium is known to cause its virulence by secreting various effector proteins directly into the host cytoplasm via two distinct type III secretion systems (T3SS-1 and T3SS-2). Generally, T3SS-1-delivered effectors help *Salmonella* Typhimurium in the early phases of infection including invasion and immune modulation of the host cells, whereas T3SS-2 effectors mainly help in the survival of *Salmonella* Typhimurium within the host cells including maintenance of *Salmonella*-containing vacuole, replication of the bacteria, and dissemination. Some of the effectors are secreted via both T3SS-1 and T3SS-2, suggesting their role in distinct phases of infection of host cells. SteA is such an effector that is secreted by both T3SS-1 and T3SS-2. It has been shown to control the membrane dynamics of the *Salmonella*-containing vacuole within the host cells in the late phases of infection. In this manuscript, toward characterizing the T3SS-1 function of SteA, we found that SteA suppresses inflammatory responses of the host by interfering with the nuclear factor kappa B pathway. Our initial observation showed that the mice infected with *steA*-deleted *Salmonella* Typhimurium (Δ*steA*) died earlier compared to the wild-type bacteria due to heightened immune responses, which indicated that SteA might suppress immune responses. Furthermore, our study revealed that SteA suppresses immune responses in macrophages by interfering with the degradation of IκB, the inhibitor of nuclear factor kappa B. SteA suppresses the ubiquitination and hence degradation of IκB by acting on Cullin-1 of the Skp-1, Cullin-1, F-box (SCF)-E3 ligase complex. Our study revealed that SteA suppresses a key step necessary for E3 ligase activation, i.e., neddylation of Cullin-1 by interfering with dissociation of its inhibitor Cand-1.

## Introduction

*Salmonella enterica* serovar Typhimurium utilizes diverse strategies to subvert host defenses by translocating various effectors via a specialized needle-like complex called the type III secretion system (T3SS) (1, 2). The *Salmonella* T3SSs are encoded by small stretches of chromosomes known as *Salmonella* pathogenicity island-1 (SP-1; encoding T3SS-1) and SPI-2 (encoding T3SS-2) (2–5). The T3SS effectors are either encoded or regulated by the SPI-1 or SPI-2 (6). The effectors modulate different functions of the host and help the bacteria to invade, survive, and replicate in the host cell (5–9).

Before the *Salmonella* enters into the host cell, the T3SS-1 is assembled by the bacteria across the host cell membrane. It generally translocates effectors, which are required for invasion and modulation of host immune responses. T3SS-1 effectors, such as SipA and SipC, bind to actin and help the bacteria in invasion (10–12); SopE and SopE2 modulate both actin rearrangement and host immune responses (13–15). Upon invasion, *Salmonella-*containing vacuoles (SCVs) are formed inside the host cell cytoplasm. Within the SCVs, bacteria replicate. T3SS-2 is formed across the SCV membrane, and the effectors are secreted into the host cell cytoplasm. T3SS-2 effectors are majorly required for maintenance of SCV and replication and spread of the bacteria (4, 7). For example, SifA helps in the formation of *Salmonella*-induced filaments (SIF) (16–18). SIFs connect the SCV to various organelles like ER, Golgi, etc. of the cell, thereby expanding the replicative niche and acquiring nutrients for the replication of *Salmonella* (19–21).

In addition, some of the effectors such as SpvD, SlrP, SteE, SteB, GtgE, etc. are regulated by both SPI-1 and SPI-2 and translocated by both T3SS-1 and T3SS-2, indicating their role in both early and later phases of infection (22, 23). SteA is one such effector molecule (24). Using Nramp1 mice, Lawley et al. have shown that SteA might play a role in the replication of *Salmonella* (25). Geddes et al. have reported that SteA could localize in the trans-Golgi network (22). However, Van Engelenburg and Palmer have later indicated that SteA could localize in *Salmonella*-induced tubules enriched with the trans-Golgi protein GalT (26). Furthermore, Domingues et al. have reported that, in the later phases of infection, SteA binds to phosphatidylinositol 4-phosphate [PI(4)P] to control membrane dynamics of SCV, thereby helping *Salmonella* to localize within SCV and in *Salmonella*-induced tubules (27, 28). Matsuda et al. have indicated that SteA could induce T3SS-1-independent inflammation and cytotoxicity in macrophages (29). McQuate et al. have reported that SteA is important for establishing infection in the early phase and in the prevention of the clearance of *Salmonella* from macrophages (30). However, the SPI-1-regulated role of SteA in *Salmonella* infection is yet to be fully established.

In this study, toward unraveling the T3SS-1 role of SteA, we have shown that SteA suppresses the proinflammatory responses induced due to *Salmonella* Typhimurium infection. We observed that SteA interferes with the activation of nuclear factor kappa B (NF-κB), which is one of the major transcription factors known to be involved in the generation of proinflammatory responses. NF-κB in its inactive state remains in the cytoplasm bound with its inhibitor IκB. IκB degradation is necessary for NF-κB activation and is mediated by its ubiquitination. The E3 ligase is responsible for the ubiquitination of IκB (31). Our study revealed that SteA inhibits activation of Cullin-1, a component of E3 ligase complex and hence ubiquitination and degradation of IκB.

## Materials and Methods

### Ethics Statement

All animal experiments were carried out in accordance with the guidelines of the Committee for the Purpose of Control and Supervision of Experiments on Animals (No. 1842/GO/ReBiBt/S/15/CPCSEA). All the protocols for animal handling were approved by the Institutional Animals Ethics Committee of Indian Institute of Science Education and Research, Mohali (IISERM/SAFE/PRT/2016-2018/004, 010, 015).

### Bacterial Strains

*S. enterica* serovar Typhimurium SL1344 strain was a kind gift from Dr. Mahak Sharma (IISER Mohali). SteA in the genome of *Salmonella* Typhimurium was replaced with a Kanamycin cassette by one-step inactivation method following the protocol by Datsenko and Warner (32). Briefly, Kanamycin cassette was amplified from the plasmid pKD13 (a kind gift from Dr. Rachna Chaba, IISER Mohali) using primers—SteA H1P2 and SteA H2P1 (Table 1). The amplified Kanamycin cassette with flanking regions corresponding to the flanking regions of the *steA* gene in the *Salmonella* Typhimurium genome was transformed into *Salmonella* Typhimurium expressing the λ-red recombinase via the helper plasmid pKD46 (a kind gift from Dr. Rachna Chaba, IISER Mohali). The colonies were selected on Kanamycin plates and screened by colony PCR. The deletion mutant of Δ*steA* was then transduced to a clean background (*Salmonella* Typhimurium SL1344) using P22 phage (a kind gift from Dr. Rachna Chaba, IISER Mohali). For complementation, *steA* gene of *Salmonella* Typhimurium (www.ncbi.nlm.gov.in) was cloned in pACYC177 (a kind gift from Dr. Rachna Chaba, IISER Mohali) using restriction cloning and was transformed in the deletion mutant of SteA (Δ*steA*) background. The Δ*spi2 Salmonella* Typhimurium 14028 (SV6017) strain was a kind gift from Dr. Francisco Ramos-Morales (University of Seville, Spain) (33). The P22 phage transduction method was used to construct Δ*spi2* SL1344 and Δ*spi2*Δ*steA* SL1344 strains. The primers used in this study are listed in Table 1, and the strains and plasmids used in this study are listed in Table 2.

**Table 1:** Primers used in this study.

**Name of primer**		**Sequence 5^**′**^-3^**′**^**
SteA H1P2		AGTCTGATTTCTAACAAAACTGGCTAAACATAAACGCTTTATTCCGGGGATCCGTCGACC
SteA H2P1		GACATATAAAGCTATTGAGCAAAATTTGAAGGAGTAGGATATGTGTAGGCTGGAGCTGCTTCG
SteA	Forward	GCGC**CATATG**ATGCCATATACATCAGTTTC
	Reverse	CGCG**GGATCC**TTAATAATTGTCCAAATAGT
SteA-GST	Forward	ATTGTT**GGATCC**CCATATACATCAGTTTCTAC
	Reverse	GTTATT**CTCGAG**TTAATAATTGTCCAAATAGTTATG
SteA-HA	Forward	CCATATT**GGATCC**CCACCATGCCATATACATCAGTTTCTACC
	Reverse	AAGCTAT**CTCGAG**TTAAGCGTAATCTGGAACATCGTATGGGTAATAATTGTCCAAATAGTTATGGTAGCGAG
SteA compl	Forward	ACCT**GGATCC**AAGCAGCATAAGATCAGGCCG
	Reverse	CGT**GACGTC**TTAATAATTGTCCAAATAGTTATGG
SteA compl-H	Forward	ACCT**GGATCC**AAGCAGCATAAGATCAGGCCG
	Reverse	CGT**GACGTC**TTAGTGATGATGATGATGATGATAATTGTCCAAATAGTTATGG
TNFα	Forward	CCCTCACACTCAGATCATCTTCT
	Reverse	GCTACGACGTGGGCTACAG
IL-6	Forward	TAGTCCTTCCTACCCCAATTTCC
	Reverse	TTGGTCCTTAGCCACTCCTTC
IκB	Forward	TTACT**GGATCC**ATGTTTCAGCCAGCTGGGCAC
	Reverse	TCGGTC**GTCGAC**TTATAATGTCAGACGCTGGCC
Rbx-1	Forward	TTACT**GGATCC**ATGGCGGCGGCGATGGATGT
	Reverse	TCGGTC**GTCGAC**CTAATGCCCATACTTCTGGAA
Skp-1	Forward	TTACT**GGATCC**ATGCCTACGATAAAGTTGCAG
	Reverse	TCGGTC**GTCGAC**TCACTTCTCTTCACACCATT
Cullin-1	Forward	CACCATGTCATCAAACAGGAGTCAGAAT
	Reverse	TCGGTC**GTCGAC**TTAAGCCAAGTAACTGTAGGT
SteA Y2H	Forward	ATTGTT**GGATCC**CCATATACATCAGTTTCTAC
	Reverse	GTTATT**GTCGAC**TTAATAATTGTCCAAATAGTTATG

*The highlighted regions indicate the restriction sites.

**Table 2:** Strains and plasmids used in this study.

**Strains *Salmonella* Typhimurium**	**Genotype or description**	**Reference**
wt	Wild-type SL1344	A kind gift from Dr. Mahak Sharma
Δ*steA*	*steA*::Km^R^	This study
compl	*steA*::Km^R^, pACYC177-*steA*	This study
compl-H	*steA*::Km^R^, pACYC177-*steA*-His	This study
14028 Δ*spi2* (SV6017)	*spi2*::Cm^R^	A kind gift from Dr. Francisco Ramos-Morales
SL1344 Δ*spi2*	*spi2*::Cm^R^	This study
Δ*spi2*Δ*steA*	*spi2*::Cm^R^, *steA*::Km^R^	This study
**Plasmids**	**Description**	**Reference**
pKD13	Template used for amplifying Kanamycin Cassette	A kind gift from Dr. Rachna Chaba
pKD46	Plasmid expressing λ-red recombinase	A kind gift from Dr. Rachna Chaba
pACYC177	Bacterial expression plasmid used for complementation	A kind gift from Dr. Rachna Chaba
pACYC177-*steA*	*steA* with its native promoter cloned in pACYC177	This study
pACYC177-*steA*-His	His-tagged *steA* with its native promoter cloned in pACYC177	This study
pGEX4T3	Plasmid expressing GST	A kind gift from Dr. Mahak Sharma
pGEX4T3-*steA*	Plasmid expressing GST-tagged SteA	This study
pcDNA3.1(+)	Mammalian expression plasmid	A kind gift from Dr. Kausik Chattopadhyay
pcDNA3.1(+)*steA*	Mammalian expression plasmid expressing HA-tagged SteA	This study
pGL4.32	NF-κB promoter reporter plasmid	Promega, USA
pRL	Plasmid expressing renilla luciferase	A kind gift from Dr. Rajesh Ramachandran
pGADC1	Yeast expression plasmid with activation domain (AD)	A kind gift from Dr. Shravan Mishra
pGADC1-*IκB*	Yeast expression plasmid with activation domain (AD) fused with IκB	This study
pGADC1-*Rbx1*	Yeast expression plasmid with activation domain (AD) fused with Rbx-1	This study
pGADC1-*Skp1*	Yeast expression plasmid with activation domain (AD) fused with Skp-1	This study
pGADT7	Yeast expression plasmid with activation domain (AD)	A kind gift from Dr. Ram K. Yadav
pGADT7-*Cullin1*	Yeast expression plasmid with activation domain (AD) fused with Cullin-1	This study
pGBDUC1	Yeast expression plasmid with binding domain (BD)	A kind gift from Dr. Shravan K. Mishra
pGBDUC1-*steA*	Yeast expression plasmid with binding domain (BD) fused with SteA	This study

### Cell Lines and Culture Conditions

RAW 264.7 (a murine macrophage cell line) used in this study was obtained from the National Center for Cell Science, Pune, India, and HEK 293 (a human kidney epithelial cell line) was obtained from American Type Culture Collection. The cells were maintained, respectively, in Roswell Park Memorial Institute (RPMI) 1640 or Dulbecco's modified Eagle medium (DMEM) supplemented with 10% (*v*/*v*) fetal bovine serum (FBS), 100 U/ml of penicillin, and 100 μg/ml of streptomycin (Invitrogen, Life Technologies, USA) at 37°C and 5% CO_2_.

### Differentiation of Bone Marrow Cells to Bone-Marrow-Derived Macrophages

Balb/c mice 6–8 weeks old were euthanized, and their femur and tibia bones were extracted. The muscle tissue of the bones was then removed off and were then washed with ice-cold phosphate-buffered saline (PBS). They were then dipped in 70% (*v*/*v*) alcohol for 2 min and were transferred to RPMI 1640 media. Then, using sterile scissors, the epiphyses of the bones were cut, and the bone marrow cells were extracted by flushing the bones with RPMI 1640 media. These bone marrow cells were then differentiated to bone-marrow-derived macrophages (BMDMs) using macrophage colony-stimulating factor (M-CSF). Briefly, cells were suspended in differentiation media [RPMI 1640 supplemented with 10% (*v*/*v*) FBS, 100 U/ml of Penicillin, 100 μg/ml of Streptomycin, 1 mM sodium pyruvate, 0.1 mM nonessential amino acids, 1% (*v*/*v*) β-mercaptoethanol, and 20 ng/ml of M-CSF] and plated in 24-well plates. They were incubated at 37°C with 5% CO_2_. The media were changed every 2 days, and fresh differentiation media was added. The adhered cells obtained at day 7 were BMDMs and were used for further experiments.

### Infection of Cells

For inducing SPI-1 conditions, as described previously by Cardenal-Munoz and Ramos-Morales (24), bacteria were grown to stationary phase in Luria–Bertani (LB) broth supplemented with 0.3 M NaCl and appropriate antibiotics. Cells were plated in 24- or 6-well plates at a density of 1 × 10^6^ cells/ml for overnight and infected with the stationary phase bacteria at a multiplicity of infection (MOI) of 10:1 (for BMDMs) or 20:1 (for RAW 264.7 and HEK 293 cells) for 30 min. The media containing the bacteria was removed, and fresh media supplemented with 100 μg/ml of gentamicin were added to the wells. After 1 h, this media was replaced with fresh media supplemented with 20 μg/ml of gentamicin for different time points depending on the assay. In all the experiments, the invasion was checked after 2 h of infection by enumerating the bacteria after the cells were lysed with 0.1% (*v*/*v*) Triton-X-100 (Himedia, India) in PBS.

### Cell Cytotoxicity Assay

RAW 264.7 and BMDMs were plated at a density of 1 × 10^6^ cells/ml and infected with wild type (wt), Δ*steA*, or compl strains at an MOI of 20:1 or 10:1, respectively. After 8 h of infection, supernatants were collected, and cell cytotoxicity was checked using lactate dehydrogenase release assay (Promega, USA), according to the manufacturer's protocol.

### Mice Infection and Scoring

Bacteria grown overnight in LB medium supplemented with 50 μg/ml of streptomycin were subcultured and grown until log phase at 37°C. Balb/c mice 6–8 weeks old were infected intraperitoneally with 5 × 10^5^ or 5 × 10^7^ log-phase bacteria, and the survival of mice was monitored for 24 and 96 h, respectively. *p* values were calculated using Kaplan–Meir test using the software R. In addition, 36 h postinfection (hpi), the mice infected with 5 × 10^5^ bacteria were scored for their response to stimuli, the extent of decrease in activity, the extent of eye closure and the pilorected fur as compared to the uninfected mice as described previously by Shrum et al. (34). In addition, the core body temperature of mice was checked using a noncontact infrared thermometer at 24 h after infection with 5 × 10^5^ bacteria. The body temperature of mice was also checked before infection, and the difference in temperature of each mice was calculated. The blood of infected mice was collected at 24 and 36 h after infection, and serum was isolated from it. The serum was analyzed for tumor necrosis factor alpha (TNFα), interleukin (IL)-6, IL-1β, interferon gamma (IFN-γ), IL-12, and IL-10 (BD Biosciences, USA) using ELISA according to manufacturer's protocol.

### Colonization and Splenic Lysate Preparation

Balb/c mice 6–8 weeks old were infected intraperitoneally with 5 × 10^5^ log-phase bacteria. At 36 hpi, spleens were isolated and homogenized in PBS using a sterile pestle.

For colonization, the homogenate was subjected to lysis with 0.1% (*v*/*v*) Triton-X-100 at 37°C for 30 min. The bacteria were enumerated after serial dilution on LB agar (LA) plates supplemented with 50 μg/ml of streptomycin.

For splenic lysate preparation, the homogenate was pelleted by centrifugation at 2,000×*g* for 5 min and washed twice with PBS. Then, the pellet was treated with 500 μl of ACK lysis buffer [154.95 mM NH_4_Cl, 10 mM KHCO_3_, and 0.1 mM ethylenediaminetetraacetic acid (EDTA); pH 7.2] at room temperature for 5 min. To stop the reaction, 100 μl of FBS was added to it and was centrifuged at 2,000×*g* for 5 min and washed twice with PBS. The pellet was then resuspended in 70 μl of RIPA buffer [50 mM Tris–Cl (pH 8), 150 mM NaCl, 5 mM EDTA, 1% (*v*/*v*) NP-40, 0.5% (*w*/*v*) sodium deoxycholate, and 0.1% (*w*/*v*) sodium dodecyl sulfate] containing 1× mammalian protease inhibitor cocktail (Sigma-Aldrich, USA) and sonicated at 10 Å for three pulses of 5 s each. This was then centrifuged at 24,000×*g* for 30 min. The supernatant thus obtained was the splenic lysate.

### TNFα and IL-6 Gene Expression

Balb/c mice 6–8 weeks old were infected intraperitoneally with 5 × 10^5^ log-phase bacteria. At 36 hpi, spleens were isolated and were subjected to ballooning with 400 U/ml Collagenase D in HBSS and incubated at 37°C for 25 min. To stop the reaction, 100 μl of 0.5 M EDTA was added and incubated at 37°C for 5 min. This was then passed through a 40-μm strainer, and 5 ml of ice-cold RPMI 1640 media supplemented with 10% (*v*/*v*) FBS was added to it. The cells were then harvested at 2,000×*g* for 5 min and washed with PBS. The pellet was resuspended in 3 ml of 30% bovine serum albumin (Sigma-Aldrich, USA). To this, 1 ml of PBS was added slowly along the wall of the conical tube to form a layer and was subjected to density gradient centrifugation (with zero acceleration or deceleration) at 2,200×*g* for 30 min at 12°C. The interface of the two layers contained the mononuclear cells, which were then harvested, and RNA was isolated according to the manufacturer's protocol using the Nucleopore RNA isolation Kit (Genetix, India). The complementary DNA (cDNA) was then synthesized using Verso-cDNA kit (Thermo-Fisher, USA). The PCRs were performed using Maxima SYBR green qPCR master mix (Thermo-Fisher, USA) and the Eppendorf Realplex master cycler. Primer sequences were used from the Harvard primer bank and were synthesized by Integrated DNA Technologies, USA.

### Quantification of TNFα

RAW 264.7 and BMDMs were plated at a density of 1 × 10^6^ cells/ml and infected with wt, Δ*steA*, or compl strains at an MOI of 20:1 or 10:1, respectively. The supernatant was collected after 8 h of infection, and TNFα was quantified using ELISA (BD Biosciences, USA) as per the manufacturer's protocol. Furthermore, to confirm equal invasion in all the experiments, cells were lysed after 2 h of infection with 0.1% (*v*/*v*) Triton-X-100 at 37°C for 30 min, and bacteria were enumerated on LA plates.

### Transfection of HEK 293 Cells

HEK 293 (a human kidney epithelial cell line) cells (7 × 10^6^) were plated in DMEM in a 90-mm Petri dish for whole-cell lysate preparation, and 2.5 × 10^4^ HEK 293 cells were seeded on coverslips in a 24-well plate for colocalization studies. Cells were then transfected with 3 μg (for whole-cell lysates) or 1 μg (for colocalization) of pCDNA3.1(+) or pCDNA3.1(+)*steA* (Table 2) using polyethyleneimine at a ratio of 1:3 (DNA/polyethyleneimine). The media was changed after 8 h of transfection. Then, after 24 or 36 h of transfection, the cells were stimulated with 10 ng/ml of TNFα for 15 or 30 min for whole-cell lysate preparation and 30 min for colocalization studies.

### Luciferase Reporter Assay

#### For HEK 293 Cells

HEK 293 cells (2.5 × 10^4^) were plated in 100 μl of DMEM in a 96-well plate and were transfected with 0.1 μg each of NF-κB reporter plasmid pGL4.1 (Promega, USA) and renilla luciferase plasmid (pRL) (Table 2) using Lipofectamine 3000 (Promega, USA) as per the manufacturer's protocol. In addition, in assays for studying the effect of endogenous expression of SteA on NF-κB activation, cells were also transfected with pcDNA3.1(+) or pcDNA3.1(+)*steA* in addition to pGL4.32 and pRL. After 18 h of transfection, the cells were either infected with wt, Δ*steA*, and compl or stimulated with 10 ng/ml of TNFα for 6 and 8 h, respectively.

#### For RAW 264.7 Cells

RAW 264.7 cells (5 × 10^4^) were plated in 100 μl of RPMI in a 96-well plate and were transfected with 0.15 μg each of NF-κB reporter plasmid pGL4.1 (Promega, USA) and pRL (Table 2) using FuGene HD (Promega, USA) as per the manufacturer's protocol. After 12 h of transfection, the cells were infected with wt, Δ*steA*, and compl or stimulated with 500 ng/ml of LPS for 15 h.

The dual luciferase assay kit (Promega, USA) was used according to the manufactures' protocol to analyze the luminescence corresponding to NF-κB activation (firefly luciferase) and the renilla luciferase. The luminescence was detected using a plate reader (BMG Biotech, Germany). The luminescence corresponding to NF-κB activation was normalized to that of renilla luciferase (corresponding to the transfection efficiency). In infection-based experiments, the luminescence was also normalized to the invasion.

### Colocalization Studies

After 24 h of transfection in HEK 293 cells and TNFα stimulation for 30 min (as described previously), cells were washed twice with PBS and were then fixed with 2.5% (*w*/*v*) of paraformaldehyde for 30 min at room temperature. Cells were then washed twice with PBS and incubated with anti-IκB (1:750) (Santa Cruz Biotechnologies, USA), anti-HA (1:250) (Biolegend, USA), anti-Cullin-1 (1:250) (Cell Signaling Technologies, USA), or anti-Cand-1 (1:500) (Cell Signaling Technologies, USA) antibodies for 45 min at room temperature. Then, cells were washed thrice with PBS and incubated with Alexa 488-conjugated anti-rabbit immunoglobulin G secondary antibody (1:500) (Life Technologies, USA) and Alexa 568-conjugated anti-mouse immunoglobulin G secondary antibody (1:500) (Life Technologies, USA) for 30 min at room temperature. All the antibody dilutions were prepared in 0.2% (*w*/*v*) Saponin dissolved in PBS. The cells were washed thrice with PBS and were mounted on glass slides using Fluoromount (Sigma-Aldrich, USA). The imaging was performed using a Zeiss confocal microscope (for colocalization of IκB or Cullin-1 with SteA-HA) or Leica confocal microscope (for colocalization of Cand-1 with SteA-HA).

### Whole-Cell Lysate Preparation

#### For Infection

Cells were plated at a density of 1.5 × 10^6^/ml in a six-well plate for overnight. Cells (4.5 × 10^6^) (three wells of 1.5 × 10^6^/ml) were infected with wt, Δ*steA*, or compl for 30 min. Then, the bacteria were removed, and RPMI 1640 media containing 100 μg/ml of gentamicin was added to each well for 30 min. The cells were then washed twice with sterile PBS and were then harvested at 2,000×*g* for 5 min. The pellet was then resuspended in 70–100 μl of whole-cell lysis buffer [50 mM Tris–Cl, 150 mM NaCl, 0.1% (*w*/*v*) sodium dodecyl sulfate (SDS) and 0.1% (*v*/*v*) Triton-X-100, pH 8] with mammalian protease inhibitor cocktail (Sigma-Aldrich, USA) and subjected to sonication at 10 Å for 15 s with a pulse of 5 s each. Then, it was centrifuged at 16,000×*g* for 30 min at 4°C. The supernatant thus obtained was the whole-cell lysate. In addition, the invasion was also checked by colony-forming unit counting for all the experiments.

#### For Transfections

After 24 h of transfection in HEK 293 cells (as described previously), cells were washed twice with PBS and were harvested by centrifugation at 2,000×*g* for 5 min. The pellet was then resuspended in 150–200 μl of whole-cell lysis buffer supplemented with mammalian protease inhibitor cocktail. This was then subjected to sonication at 10 Å for 15 s with a pulse of 5 s each. Then, it was centrifuged at 16,000×*g* for 30 min at 4°C, and the supernatant obtained was collected as whole-cell lysate.

### Nuclear Lysate Preparation

Cells (4.5 × 10^6^) (three wells of 1.5 × 10^6^/ml) of RAW 264.7 were plated in a six-well plate overnight for each sample and then infected with wt, Δ*steA*, or compl for 30 min. Then, the bacteria were removed, and RPMI 1640 media containing 100 μg/ml of gentamicin were added to each well for 30 min. Cells were then washed twice with sterile PBS and were then harvested at 2,000×*g* for 5 min. The pellet volume was measured, and it was dissolved in five times the pellet volume in hypotonic buffer (10 mM HEPES pH 7.9 with 1.5 mM MgCl_2_ and 10 mM KCl) and centrifuged at 1,850×*g* for 5 min at 4°C. Then, the pellet was dissolved in hypotonic buffer with 0.5 M dithiothreitol and mammalian protease inhibitor and was then incubated on ice for 15 min. This was then sonicated at 10 Å for 15 s with three pulses of 5 s each and centrifuged at 3,300×*g* for 15 min at 4°C. The pellet was then resuspended in 70 μl of low salt buffer [20 mM HEPES pH 7.9, 1.5 mM MgCl_2_, 20 mM KCl, 0.2 mM EDTA, and 25% (*v*/*v*) glycerol] with 0.5 M DTT and mammalian protease inhibitor. To this, 30 μl of high salt buffer [20 mM HEPES pH 7.9, 1.5 mM MgCl_2_, 800 mM KCl, 0.2 mM EDTA, and 25 % (*v*/*v*) glycerol] was added dropwise and incubated on ice for 10 min before subjecting to sonication at 10 Å for 15 s. Then, it was incubated on ice for 30 min with periodic shaking and centrifuged at 24,000×*g* for 30 min at 4°C. The supernatant hence obtained contained the nuclear lysate.

### Immunoblotting

The whole-cell lysates or the nuclear lysates were run on SDS-polyacrylamide gel electrophoresis (SDS-PAGE) gel and were transferred to polyvinylidene difluoride membrane using wet transfer. After the transfer, the membrane was incubated with 5% (*w*/*v*) bovine serum albumin (BSA) in TBST [20 mM Tris buffer pH 7.5, 150 mM NaCl, and 0.1% (*v*/*v*) Tween 20] for blocking. Then, it was incubated with various antibodies purchased from different companies. The antibodies against IκB, glyceraldehyde 3-phosphate dehydrogenase (GAPDH), p65, c-Rel, and Lamin B1 were purchased from Santa Cruz Biotechnologies, USA. The antibodies against p-p38, p38, phosphorylated c-Jun N-terminal kinase (p-JNK), JNK, IκB kinase α/β (IKKα/β), Cullin-1, Cand-1, β-TrCP, Skp-1 and Rbx-1 were purchased from Cell Signaling Technologies, USA. The antibodies against His-tag, HA-tag, and proliferating cell nuclear antigen (PCNA) were purchased from Biolegend, USA. The antibody against Nedd8-Cullin was purchased from Abcam, UK. The antibody against LAMP-1 was purchased from Thermo Fisher Scientific, USA.

The horseradish-peroxidase-tagged secondary antibodies and antibody against glutathione-S-transferase (GST) were purchased from Sigma-Aldrich, USA. The immunoblots were developed using Clarity^TM^ ECL Substrate (Bio-Rad, USA) and detected using LAS 4000 (GE Healthcare Technologies, USA).

### Densitometric Analysis

The densitometric analysis of the immunoblots was carried out using the Image J software. The band intensities of IκB, IKKα/β, p65, or c-Rel were normalized to the respective band intensities of the loading controls, i.e., glyceraldehyde 3-phosphate dehydrogenase for whole-cell lysates and PCNA for nuclear lysates. The band intensities of phosphorylated p38 and JNK were normalized to the band intensities of the total p38 and JNK. The lane intensities of ubiquitination and the band intensities of Cand-1 were normalized to the band intensities of IκB, and the band intensities of neddylated Cullin was normalized to the band intensities of Cullin-1.

### Coimmunoprecipitation Studies

Whole-cell lysates were prepared (as previously described) and were incubated with 1–2 μg of anti-HA, anti-His, anti-Cullin-1, or anti-IκB antibody for 3–4 h with continuous low-speed shaking at 4°C. Then, 20 μl of Protein A/G beads (Santa Cruz Biotechnologies, USA) was added, and it was subjected to continuous low-speed shaking at 4°C for overnight. Then, the beads were washed thrice with whole-cell lysis buffer by centrifugation at 6,000×*g* for 5 min at 4°C. The beads were resuspended in SDS-loading buffer and boiled for 10 min. The samples were then run on SDS-PAGE gel and subjected to immunoblotting.

#### For IκB Pulldown

HEK 293 cells were transfected with 3 μg of pcDNA3.1(+) or pcDNA3.1(+)*steA* (as described previously). After 24 h of transfection, cells were treated with 20 nM of proteasomal inhibitor, MG132 (Sigma-Aldrich, USA), for 3 h. The cells were then stimulated with 10 ng/ml of TNFα for 20 min, and whole-cell lysates were prepared. The whole-cell lysates were then subjected to coimmunoprecipitation with 2 μg of the anti-IκB antibody as described above.

### GST Pulldown Assay

The *steA* gene was cloned in the plasmid pGEX4T3 (Table 2) using restriction cloning. The empty pGEX4T3 and pGEX4T3-*steA* were each transformed to *Escherichia coli* Origami B1 cells (Merck, Germany) by chemical transformation method. Then, using these transformants, GST and GST-tagged SteA were overexpressed using 0.1 mM isopropyl β-d-1-thiogalactopyranoside at 18°C for 24 h. The bacteria were pelleted at 2,050×*g* for 30 min at 4°C. The pellet was resuspended in PBS containing bacterial protease inhibitor cocktail (Sigma-Aldrich, USA) and was subjected to sonication at 25 Å for 15 min. Then, this was centrifuged at 18,500×*g* for 50 min at 4°C and analyzed by SDS-PAGE. Both GST and GST-tagged SteA were found to be present in the soluble fractions. Parallelly, the glutathione beads (Qiagen, USA) were washed twice with lysis buffer [50 mM Tris–Cl, 150 mM NaCl, 0.05% (*v*/*v*) NP-40, pH 7.5]. Then, to 10 ml of the soluble fraction containing GST or GST-tagged SteA, 300 μl of washed glutathione beads were added and incubated at 4°C with shaking. After 2 h of incubation, the beads were pelleted at 1,800×*g* for 5 min and washed four times with lysis buffer. Then, the beads were incubated with 500 μl of 5% (*w*/*v*) BSA in PBS for 90 min. Then, the beads were pelleted and washed four times with lysis buffer. To this, whole-cell lysate of unstimulated RAW 264.7 cells was added and incubated at 4°C for 3–4 h with shaking. Then, the beads were pelleted and washed four times with lysis buffer and resuspended in 40 μl of SDS-loading buffer. This was then boiled for 5 min and subjected to SDS-PAGE and immunoblotting.

### Yeast Two-Hybrid Assay

The coding sequences of IκB, Skp-1, Rbx-1, and Cullin-1 from *Mus musculus* were taken from www.ncbi.nlm.gov.in. A cDNA library was prepared according to manufacturer's protocol (Invitrogen, Life Technologies, USA) using RNA isolated (according to the manufacturer's protocol; Genetix, India) from untreated RAW 264.7 cells. IκB, Skp-1, and Rbx-1 were amplified from the cDNA library and cloned in pGADC1 (Table 2) plasmid by restriction cloning. Cullin-1 was amplified and cloned in pGADT7 (Table 2) by gateway cloning method according to manufacturer's protocol (Thermo-Fisher, USA). The *steA* gene was amplified from the *Salmonella* Typhimurium genome and cloned in pGBDC1 plasmid (Table 2) by restriction cloning. The cloned AD and the BD plasmids were cotransformed in yeast (PJ697a) and plated on synthetic complete media deficient in leucine and uracil (SC-Leu, SC-Ura). The colonies thus obtained were then spotted on plates with media deficient in leucine, uracil, and histidine (SC-Leu, SC-Ura, SC-His).

### Statistical Analysis

Data were expressed as mean ± standard error of mean (SEM), and *p* values were calculated using Student's two-sided *t* test or by one-way ANOVA followed by Tukey's multiple comparisons test. Significance is indicated as follows: ^*^p < 0.05, ^**^ *p* < 0.01, ^***^p < 0.001, and nonsignificant (ns) when *p* > 0.05.

## Results

### SteA Affects the Pathogenesis of *Salmonella* Typhimurium

**Figure 1:**
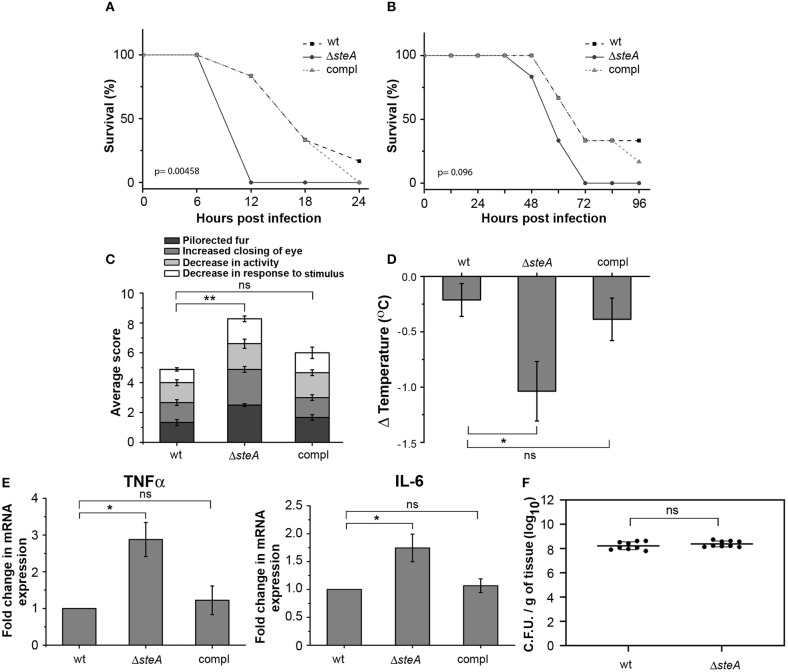
Δ*steA*-infected mice had lower survival and higher immune responses than the wt or compl. **(A,B)** Balb/c mice infected with the *steA* deletion mutant (Δ*steA*) had lower survival than those infected with wild type (wt) and the *steA*-complemented (compl) strain of *Salmonella* Typhimurium. Balb/c mice 6–8 weeks old were infected intraperitoneally with 5 × 10^7^ bacteria and monitored over a period of 24 h **(A)** or with 5 × 10^5^ bacteria and monitored over a period of 96 h **(B)**. After every 6 h **(A)** or 12 h **(B)**, the percentage of survival was calculated as (the number of mice surviving/total mice infected) × 100. The graph represents the percentage of survival of mice infected with wt, Δ*steA*, or compl strains with six mice in each group. *p* values were calculated using Kaplan–Meir survival test. **(C)** Balb/c mice infected with Δ*steA* showed advanced symptoms of a heightened immune response as compared to the wt and the compl-infected mice. Balb/c mice 6–8 weeks old were infected intraperitoneally with 5 × 10^5^ bacteria and scored for symptoms, namely, decrease in activity, decrease in response to stimuli, extent of closing of the eye, and the extent of pilorected fur at 36 h postinfection (hpi). For each experiment, the average of total scores for three or four mice infected with either wt, Δ*steA*, or compl was calculated. Bar graph represents mean ± SEM from three independent experiments. *p* values were calculated using one-way ANOVA of the total scores from three independent experiments (*p < 0.05, **p < 0.01, ***p < 0.001, ns *p* > 0.05 vs. wt-infected mice). **(D)** Body temperatures of Balb/c mice infected with Δ*steA* were lower than wt- or compl-infected mice. Balb/c mice 6–8 weeks old were infected intraperitoneally with 5 × 10^5^ bacteria, and the body temperatures of mice were recorded before infection and at 24 hpi. Then, the difference in body temperature was calculated (ΔTemperature). Bar graph represents mean ± SEM from eight mice in each group. *p* values were calculated using one-way ANOVA of ΔTemperature of eight mice from each group (*n* = 8) (*p < 0.05, **p < 0.01, ***p < 0.001, ns *p* > 0.05 vs. wt-infected mice). **(E)** Tumor necrosis factor alpha (TNFα) and interleukin (IL)-6 gene expression was higher in mononuclear cells isolated from spleen of mice infected with Δ*steA* compared to those infected with wt or compl. The spleens of Balb/c mice infected with 5 × 10^5^ bacteria were isolated at 36 hpi. The mononuclear cells were isolated using density-dependent separation [with bovine serum albumin (BSA)]. RNA was isolated from these cells; then, complementary DNA (cDNA) was prepared and gene expression of TNFα and IL-6 was analyzed by semiquantitative PCR. Fold change in gene expressions was calculated with respect to the gene expression in cells from mice infected with wt. Bar graph represents mean ± SEM from three independent experiments. *p* values were calculated using one-way ANOVA (*p < 0.05, **p < 0.01, ***p < 0.001, ns *p* > 0.05 vs. wt-infected mice). **(F)** No significant difference in the colonization of wt and Δ*steA Salmonella* Typhimurium in spleen of infected mice. Bacteria were enumerated in spleen of Balb/c mice infected intraperitoneally with 5 × 10^5^ bacteria at 36 hpi. Graph represents colony-forming units (CFU) enumerated per milligram of spleen tissue from nine mice each (represented as a dot •). *p* values were calculated using Student's *t* test (*p < 0.05, **p < 0.01, ***p < 0.001, ns *p* > 0.05 vs. wt-infected mice).

To understand the effect of SteA deletion in mouse model of *Salmonella* infection, we constructed *steA* deletion mutant (Δ*steA*) of *Salmonella* Typhimurium and *steA*-complemented strain (compl) with *steA*-containing plasmid on Δ*steA* background. Furthermore, we infected Balb/c mice with 5 × 10^5^ and 5 × 10^7^ wt, Δ*steA*, and compl *Salmonella* Typhimurium. We have observed that the mice infected with Δ*steA* died earlier than the wt and compl-infected mice (Figures 1A, B). Furthermore, we have observed that Δ*steA*-infected mice showed symptoms, namely, pilorected fur, decrease in activity, decrease in response to stimulus, and the extent of closing of the eyes that were graded on a scale of 1–4 (34) higher as compared to the control mice (Figure 1C). The scoring indicated that the mice infected with the Δ*steA* strain probably died of a heightened immune response. We also checked the body temperatures of mice infected with 5 × 10^5^ wt, Δ*steA*, and compl and found lower body temperatures of Δ*steA*-infected mice, indicating that probably mice were undergoing sepsis (35).

Furthermore, to probe whether there was any increase in the proinflammatory cytokine levels in Δ*steA*-infected mice compared to the controls, we checked TNFα, IL-6, IL-1β, IFN-γ, IL-12, and IL-10 in the serum of the infected mice and found higher TNFα, IL-6, IFN-γ, IL-12, and IL-10 level in the serum of the Δ*steA*-infected compared to wt- or compl-infected mice (Figure S1). Furthermore, we have isolated mononuclear cells from the spleen of mice infected with wt, Δ*steA*, and compl strains and analyzed the expression of TNFα and IL-6 genes. We have observed higher expression of both TNFα and IL-6 genes in cells isolated from mice infected with the Δ*steA* strains as compared to the wt- and compl-infected mice (Figure 1E). Therefore, in accordance with our hypothesis, it seemed that there was an elevated inflammatory immune response in Δ*steA*-infected mice compared to the controls. Furthermore, to check if the difference in immune response was due to a difference in the colonization pattern of Δ*steA* compared to the wt strain, we have isolated spleen and enumerated the bacteria by colony-forming unit counting. No difference in the colonization of *Salmonella* Typhimurium was observed in the spleen of these respective strain-infected mice (Figure 1F). Altogether, these results suggested that during *Salmonella* Typhimurium infection, absence of SteA enhances the host-inflammatory responses.

### Increased TNFα Production in Macrophages Infected With Δ*steA*

**Figure 2:**
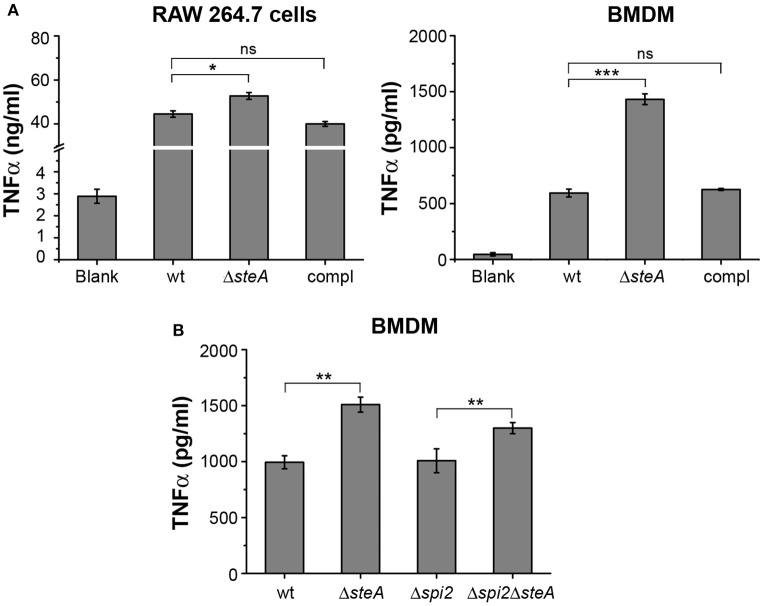
SteA suppresses proinflammatory responses in macrophages during *Salmonella* Typhimurium infection. **(A)** Tumor necrosis factor alpha (TNFα) production was higher in RAW 264.7 macrophages and bone-marrow-derived macrophages (BMDMs) infected with Δ*steA* as compared to the controls. RAW 264.7 cells and BMDMs were infected with wt, Δ*steA*, or the compl [at an multiplicity of infection (MOI) of 20:1 for RAW 264.7 and 10:1 for BMDMs]. The supernatant was collected after 8 h of infection and analyzed for TNFα by ELISA. Bar graph represents mean ± SEM from three independent experiments. *p* values were calculated using one-way ANOVA (*p < 0.05, **p < 0.01, ***p < 0.001, ns *p* > 0.05 vs. wt-infected cells). **(B)** SteA-mediated immune suppression is not affected by the absence of *spi2* gene cluster (Δ*spi2*), indicating that this effect of SteA is not SPI-2 dependent. BMDMs were infected at an MOI of 10:1 with wt, Δ*steA*, Δ*spi2*, and Δ*spi2*Δ*steA*. Supernatant was collected after 8 h, and TNFα was quantified by ELISA. Bar graph represents mean ± SEM from three independent experiments. *p* values were calculated using Student's *t* test (*p < 0.05, **p < 0.01, ***p < 0.001, ns *p* > 0.05 vs. wt or Δ*spi2*-infected cells).

To further confirm that there is heightened inflammatory response upon infection with Δ*steA*, we infected RAW 264.7 (murine macrophage cell line) and BMDMs with wt, Δ*steA*, and compl strains. After 8 h of infection, supernatants were collected and analyzed for TNFα. In both the cell types, a higher TNFα production was observed when infected with Δ*steA* compared to the wt and the compl-infected cells (Figure 2A). We further checked the cell cytotoxicity upon infection with wt, Δ*steA*, and compl strains to probe whether this difference in TNFα production was due to a difference in cell death. However, we did not find any difference in cell cytotoxicity in RAW 264.7 or BMDMs infected with wt, Δ*steA* or compl (Figure S2). Therefore, our data confirmed that the *Salmonella* Typhimurium-mediated host-immune responses increase in the absence of SteA.

SteA is secreted by both T3SS-1 and T3SS-2. In addition, recently, it was reported that SteA could be secreted by T3SS-2 as early as 15 min in RAW 264.7 cells (24). Therefore, we wanted to check whether the observed effect of SteA was due to T3SS-2 effector function. To address this, we have used a Δ*spi2* mutant of *Salmonella* Typhimurium and a Δ*steA* mutant in Δ*spi2* background (Δ*spi2*Δ*steA*). We have treated BMDMs with wt, Δ*steA*, Δ*spi2*, and Δ*spi2*Δ*steA* strains. After 8 h, we collected the supernatants and analyzed for TNFα by ELISA. The cells infected with Δ*spi2*Δ*steA* showed higher TNFα production than Δ*spi2-*infected cells in BMDMs (Figure 2B), indicating that SteA-mediated effect on the immune response is not T3SS-2 dependent.

### SteA Acts on the NF-κB Pathway to Suppress the Immune Responses

**Figure 3:**
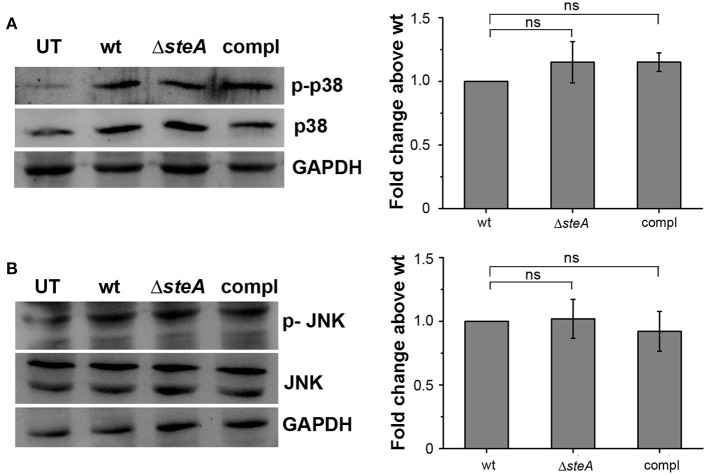
SteA does not affect the mitogen-activated protein (MAP)-kinase activation. **(A,B)** The phosphorylation levels of the MAP kinases p38 **(A)** and c-Jun N-terminal kinase (JNK) **(B)** remained unchanged upon infection with Δ*steA* as compared to the controls. RAW 264.7 cells were infected with wt, Δ*steA*, or compl strains at an multiplicity of infection (MOI) of 20:1, and whole-cell lysates were prepared after 30 min of infection. Whole-cell lysates were probed for phosphorylated levels of p38 and JNK by Western blots and densitometric analysis along with total p38 and JNK levels. Glyceraldehyde 3-phosphate dehydrogenase (GAPDH) was used as a loading control. For densitometric analysis, fold change was calculated with respect to wt. Bar graphs represent mean ± SEM from three independent experiments. *p* values were calculated using one-way ANOVA (*p < 0.05, **p < 0.01, ***p < 0.001, ns *p* > 0.05 vs. wt-infected cells). UT stands for untreated cells.

An immune response involves the activation of majorly two transcription factors NF-κB and AP-1. The phosphorylation of mitogen-activated protein (MAP) kinases p38 and/or JNK majorly leads to the activation of AP-1 (36). To check whether MAP kinases could be involved in Δ*steA*-mediated heightened immune response, we infected RAW 264.7 cells with wt, Δ*steA*, and compl strains and checked whether there is an increase in the phosphorylation levels of p38 and JNK MAP kinases in the whole-cell lysates. No difference in the phosphorylation level of p38 and JNK was observed in any of the treatments (Figures 3A,B). As phosphorylation levels are indicative of activation levels, our study indicated that SteA probably does not interfere in the MAP kinase activation pathway. As activation of the AP-1 transcription factor is dependent on MAP kinase activation, we presumed that probably SteA-mediated effect is not via the AP-1 pathway.

**Figure 4:**
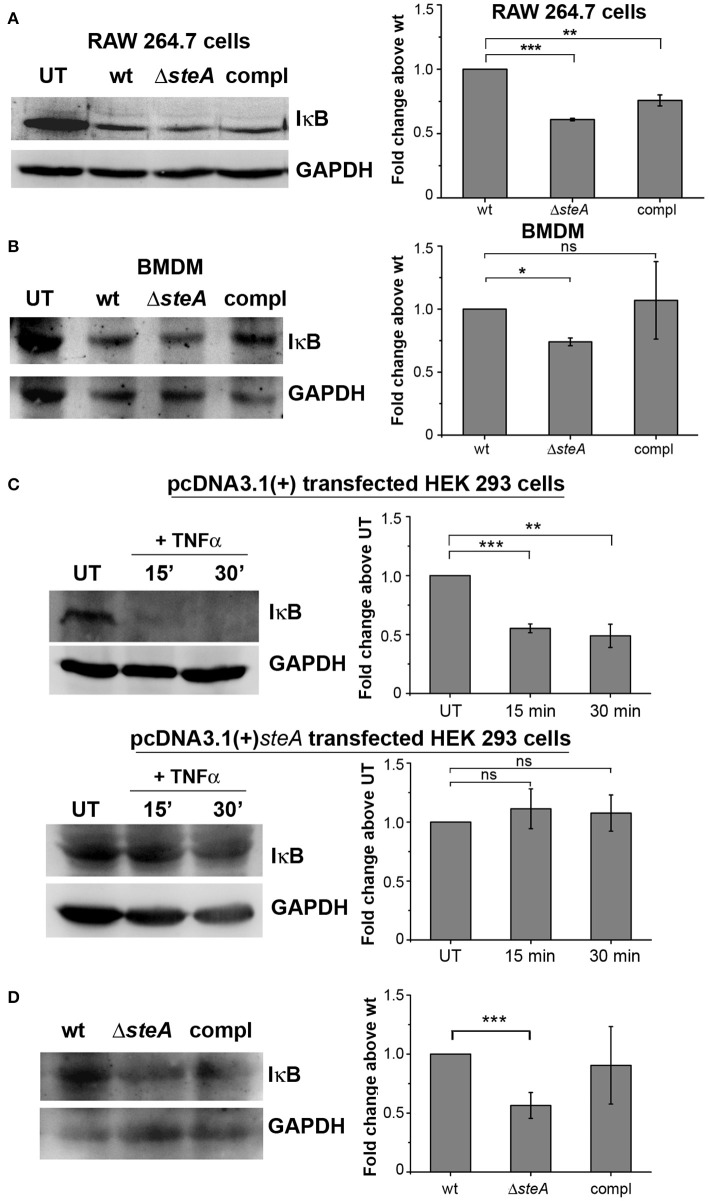
SteA hinders IκB degradation. **(A,B)** IκB degradation was higher in cells infected with Δ*steA* than the wt or compl in both RAW 264.7 cells **(A)** and bone-marrow-derived macrophages (BMDMs) **(B)** as shown by Western blot and densitometric analysis. RAW 264.7 cells and BMDMs were infected with wt, Δ*steA*, or compl at an multiplicity of infection (MOI) of 20:1 and 10:1, respectively, and whole-cell lysates were prepared after 30 min of infection. The levels of IκB were probed by Western blotting. **(A,B)** Glyceraldehyde 3-phosphate dehydrogenase (GAPDH) was used as a loading control in all the experiments. UT stands for untreated cells. For densitometric analysis, fold change was calculated with respect to wt. Bar graphs represent mean ± SEM from three independent experiments. *p* values were calculated using one-way ANOVA (*p < 0.05, **p < 0.01, ***p < 0.001, ns *p* > 0.05 vs. wt-infected cells). **(C)** Endogenous expression of SteA suppresses the degradation of IκB in HEK 293 cells. HEK 293 cells were transfected with pcDNA3.1(+)*steA* or pcDNA3.1(+) empty plasmid. After 18 h of transfection, cells were stimulated with TNFα for 15 and 30 min. Whole-cell lysates were then prepared, and IκB levels were analyzed by Western blotting and densitometry. GAPDH was used as a loading control in all the experiments. UT stands for untreated cells. For densitometric analysis, fold change was calculated with respect to UT cells. Bar graphs represent mean ± SEM from three independent experiments. *p* values were calculated using Student's *t* test (*p < 0.05, **p < 0.01, ***p < 0.001, ns *p* > 0.05 vs. UT cells). **(D)** IκB levels were lower in splenic lysates of mice infected with Δ*steA* as compared to the control-infected mice. Balb/c mice were infected with wt, Δ*steA*, or compl, and spleen was isolated at 36 hpi. Splenic lysates were then prepared, and the levels of IκB were probed by Western blotting and densitometric analysis. GAPDH was used as a loading control in all the experiments. For densitometric analysis, fold change was calculated with respect to wt. Bar graphs represent mean ± SEM from three independent experiments. *p* values were calculated using Student's *t* test (*p < 0.05, **p < 0.01, ***p < 0.001, ns *p* > 0.05 vs. wt-infected mice).

Furthermore, we wanted to check whether SteA has any effect on the activation of NF-κB. In an inactivated state, NF-κB remains bound to its inhibitor IκB, which prevents the translocation of NF-κB to the nucleus. Upon phosphorylation of IκB by upstream kinase IKKα/β, IκB gets ubiquitinated and degraded through proteasome rendering NF-κB free to translocate to the nucleus (31, 37). We observed that in Δ*steA*-infected RAW 264.7 cells and BMDMs, there is increased IκB degradation compared to control infected cells (Figures 4A, B). NF-κB is known to be activated in HEK 293 cells in response to TNFα. To confirm the observation that SteA affects the degradation of IκB, we endogenously expressed SteA in HEK 293 cells and probed the IκB levels upon stimulation with TNFα. We observed increased IκB degradation in TNFα-activated cells transfected with the empty plasmid, but in cells expressing SteA, IκB degradation was found to be inhibited (Figure 4C). Furthermore, we also observed less IκB in splenic lysates of mice infected with Δ*steA* compared to the control (Figure 4D).

**Figure 5:**
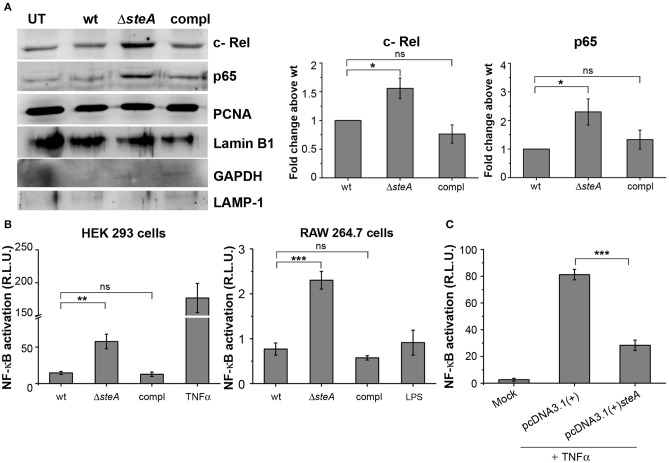
SteA suppresses the nuclear factor kappa B (NF-κB) activation. **(A)** The translocation of NF-κB subunits (p65 and c-Rel) was more in cells infected with Δ*steA* than the controls. Nuclear lysates were prepared following infection of RAW 264.7 macrophages with wt, Δ*steA*, or compl for 30 min. p65 and c-Rel were probed by Western blotting and densitometry. Proliferating cell nuclear antigen (PCNA) and Lamin B1 were used as a loading control for nuclear fractions. In addition, GAPDH and LAMP-1 were used as non-nuclear markers to check the purity of the nuclear lysates. For densitometric analysis, fold change was calculated with respect to wt. **(B)** NF-κB reporter activity was higher upon infection of HEK 293 cells and RAW 264.7 cells with Δ*steA* than the wt or the compl-infected cells. HEK 293 or RAW 264.7 cells were transfected with NF-κB luciferase reporter plasmid and pRL (renilla luciferase) plasmid. After 18 or 12 h of transfection, respectively, cells were infected with wt, Δ*steA*, or compl at an multiplicity of infection (MOI) of 20:1 (HEK 293 cells) or 5:1 (RAW 264.7 cells), and the reporter activity was measured following 8 or 15 h of infection, respectively. Tumor necrosis factor alpha (TNFα) stimulated HEK 293 cells, and lipopolysaccharide (LPS)-stimulated RAW 264.7 cells were taken as positive control for the experiment. **(A,B)** Bar graph represents mean ± SEM from three independent experiments. *p* values were calculated using one-way ANOVA (*p < 0.05, **p < 0.01, ***p < 0.001, ns *p* > 0.05 vs. wt-infected cells). **(C)** Endogenous expression of SteA in HEK 293 cells suppresses the NF-κB reporter activity upon TNFα stimulation. HEK 293 cells were transfected with NF-κB Luciferase reporter plasmid, pRL plasmid, and pcDNA3.1(+)*steA* or pcDNA3.1(+) empty plasmid. After 18 h of transfection, cells were stimulated with TNFα, and the reporter activity was measured after 8 h. Bar graph represents mean ± SEM from three independent experiments. *p* values were calculated using Student's *t* test [*p < 0.05, **p < 0.01, ***p < 0.001, ns *p* > 0.05 vs. pcDNA3.1(+) transfected cells].

More degradation of IκB suggests increased translocation of NF-κB to the nucleus. Among the NF-κB family members, p65 and/or c-Rel are involved in the transcription of proinflammatory genes (37). Thus, to probe if IκB degradation corresponds to the nuclear translocation of NF-κB, we infected RAW 264.7 cells with wt, Δ*steA*, and compl strains and checked the levels of p65 and c-Rel in the nuclear lysates. We observed that the nuclear translocation of both p65 and c-Rel were more in cells infected with Δ*steA* compared to the wt and the compl-infected cells (Figure 5A). This observation indicated that the NF-κB activation during *Salmonella* Typhimurium infection increases in the absence of SteA. Furthermore, to confirm that SteA suppresses NF-κB activation, we transfected HEK 293 and RAW 264.7 cells with NF-κB promoter-reporter plasmid and then infected with wt, Δ*steA*, and compl strains. The cells infected with Δ*steA* showed higher NF-κB reporter activity than wt and the compl strains in both HEK 293 and RAW 264.7 cells (Figure 5B). Furthermore, we wanted to know if endogenous expression of SteA could also suppress NF-κB activation. Toward this, we endogenously expressed SteA in HEK 293 cells and then transfected with the NF-κB promoter-reporter plasmid. We observed that upon stimulation with TNFα, there is diminished NF-κB reporter activity in endogenous SteA-expressing cells as compared to the cells transfected with empty plasmid (Figure 5C).

Altogether, these results confirm that SteA suppresses the immune responses by acting on the NF-κB pathway.

### SteA Interferes With the IκB Ubiquitination

**Figure 6:**
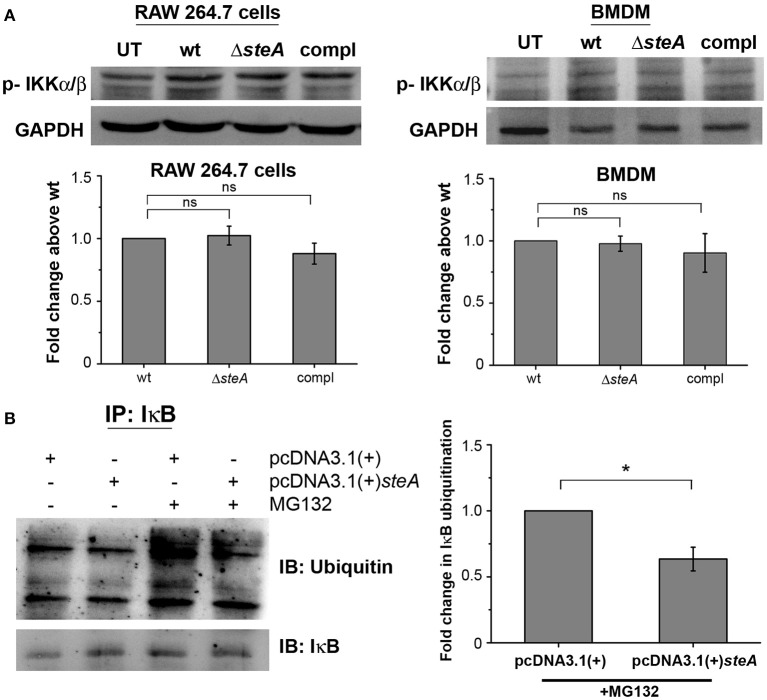
SteA acts on the ubiquitination of IκB but does not affect the activation of upstream kinase (IKK). **(A)** SteA does not affect the activation of IKKα/β. RAW 264.7 cells, and BMDMs were infected with wt, Δ*steA*, or compl at an MOI of 20:1 and 10:1, respectively, and whole-cell lysates were prepared following 30 min of infection. The levels of IKKα/β were probed by Western blotting and densitometry. For densitometric analysis, fold change was calculated with respect to wt. Bar graphs represent mean ± SEM from three independent experiments. *p* values were calculated using one-way ANOVA (*p < 0.05, **p < 0.01, ***p < 0.001, ns *p* > 0.05 vs. wt-infected cells). **(B)** SteA suppresses the ubiquitination of IκB. HEK 293 cells were transfected with pcDNA3.1(+)*steA* or pcDNA3.1(+) empty plasmid. After 24 h of transfection, cells were pretreated with proteasomal inhibitor MG132 for 3 h and stimulated with TNFα for 20 min. Whole-cell lysates were then prepared and immunoprecipitated with anti-IκB antibody. Ubiquitination was then analyzed by Western blotting and densitometry. **(A,B)** Glyceraldehyde 3-phosphate dehydrogenase (GAPDH) was used as a loading control. For densitometric analysis, fold change was calculated with respect to pcDNA3.1(+) transfected cells. Bar graphs represent mean ± SEM from three independent experiments. *p* values were calculated using Student's *t* test [*p < 0.05, **p < 0.01, ***p < 0.001, ns *p* > 0.05 vs. pcDNA3.1(+) transfected cells].

IκB phosphorylation by IKKα/β is a prerequisite for IκB degradation. In response to an infection, a signaling cascade leads to the phosphorylation of IKKα/β, which in turn phosphorylates IκB (36). Since we have observed that there is more IκB degradation in Δ*steA*-infected cells compared to the controls (Figure 4), we wanted to check whether this was due to increased activation of IKKα/β. No change was observed in the phosphorylation levels of IKKα/β in both RAW 264.7 macrophages and BMDMs infected with wt, Δ*steA*, and compl strains (Figure 6A). This result indicated that increased degradation of IκB is probably not due to increased activation of IKKα/β. Hence, SteA seems to act downstream to the activation of IKKα/β for suppression of IκB degradation.

The degradation of IκB requires its polyubiquitination. To probe if SteA was interfering with the polyubiquitination of IκB and subsequently its degradation, SteA-expressing HEK 293 cells were pretreated with a pharmacological inhibitor (MG132), which prevents the proteasomal degradation of ubiquitinylated proteins and immunoprecipitated with anti-IκB antibody. We observed that the ubiquitination of IκB was less when SteA was present (Figure 6B).

Altogether, these results showed that SteA interferes with the ubiquitination of IκB to suppress the host proinflammatory responses.

### SteA Does Not Inhibit the Recruitment of E3 Ligase Components to IκB

**Figure 7:**
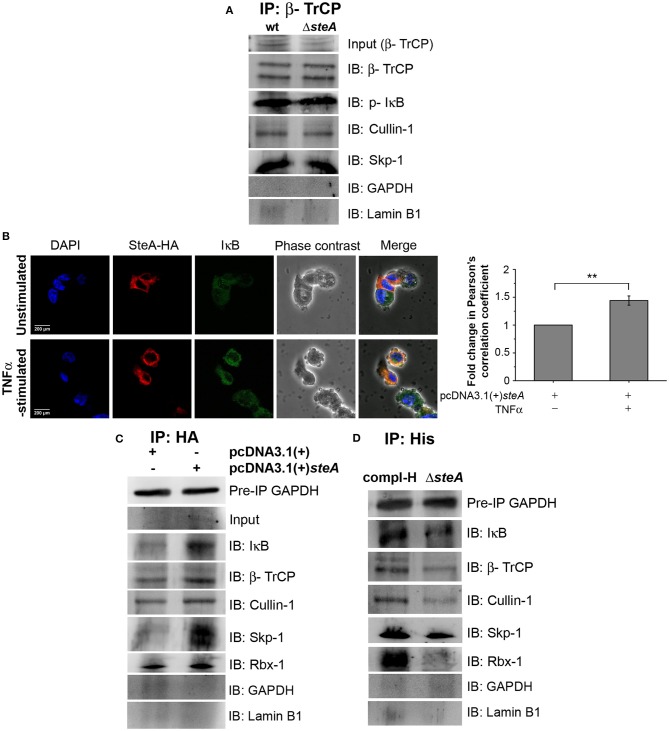
SteA localizes to the SCF-E3 ligase complex. **(A)** The SCF-E3 ligase complex assembled at phosphorylated IκB (p-IκB) in the presence or absence of SteA. RAW 264.7 cells were infected with wt or Δ*steA* strains at an multiplicity of infection (MOI) of 20:1 for 30 min. Whole-cell lysates were then prepared and subjected to immunoprecipitation with anti-β-TrCP antibody. The Skp-1, Cullin-1, F-box (SCF) complex members and p-IκB were then probed by Western blotting. Glyceraldehyde 3-phosphate dehydrogenase (GAPDH) and Lamin B1 were also probed after IP to check the specificity of the pulldowns. **(B)** The colocalization of SteA and IκB increased upon tumor necrosis factor alpha (TNFα) stimulation as compared to the unstimulated control as observed under confocal microscopy. HEK 293 cells overexpressing HA-tagged SteA were fixed after 30 min of TNFα stimulation and were incubated with anti-IκB and anti-HA primary antibodies. Then, the cells were stained with Alexa 488-tagged (for IκB), Alexa 568-tagged (for HA) secondary antibodies, and 4′,6-diamidino-2-phenylindole (DAPI; for the nucleus). The cells were then observed under a confocal microscope. The colocalization was quantified using Pearson's correlation coefficient (PCC) taking 8–10 fields per experiment. Bar graph represents as mean ± SEM from three independent experiments (*p < 0.05, **p < 0.01, ***p < 0.001, ns *p* > 0.05 vs. fold change of PCC in unstimulated cells). **(C,D)** SteA localizes to the SCF-E3 ligase complex on IκB. **(C)** HEK 293 cells were transfected with pcDNA3.1(+)*steA* or pcDNA3.1(+) empty vector. After 18 h of transfection, cells were stimulated with TNFα for 30 min, and whole-cell lysates were immunoprecipitated with anti-HA antibody. **(D)** RAW 264.7 cells were infected with Δ*steA* and Δ*steA* complemented with His-tagged SteA (compl-H) at an MOI of 20:1 for 30 min. Whole-cell lysates were then prepared and subjected to immunoprecipitation with anti-His antibody. **(C,D)** SCF complex members and IκB were then probed by Western blotting. GAPDH in the preimmunoprecipitation (pre-IP) samples was used as a loading control for the coimmunoprecipitation. GAPDH and Lamin B1 were also probed after IP to check the specificity of the pulldowns.

For ubiquitination to happen, the phosphorylated IκB is recognized by the Skp-1, Cullin-1, F-box (SCF)-E3 ligase complex. Recruitment of the SCF complex to the phosphorylated IκB happens through F-box protein β-TrCP (31, 38). Therefore, to probe whether SteA could affect the recruitment of the SCF complex to IκB, we immunoprecipitated the lysates of wt and Δ*steA*-infected RAW 264.7 macrophages with anti-β-TrCP antibody and observed the association of SCF complex with IκB both in presence or absence of SteA. This showed that SteA was not interfering with the assembly of the SCF-E3 ligase complex to phosphorylated IκB (Figure 7A and Figure S3A).

Furthermore, to understand how SteA affects IκB ubiquitination, we checked whether SteA could associate with IκB. Toward this, we overexpressed SteA in HEK 293 cells and checked the localization of SteA with IκB upon stimulation with TNFα using confocal microscopy. We observed that IκB and SteA colocalize upon stimulation in cells overexpressing SteA (Figure 7B). Furthermore, to confirm the association of SteA with IκB and SCF complex, we have immunoprecipitated HA-tagged SteA-expressing HEK 293 cell lysates using anti-HA antibody following TNFα stimulation. We have observed that the members of the SCF complex and IκB coimmunoprecipitated with SteA (Figure 7C and Figure S3B). Furthermore, upon immunoprecipitation of RAW 264.7 cells infected with Δ*steA* and complemented strain expressing His-tagged SteA (compl-H) using anti-His antibody, we observed that SteA coimmunoprecipitated with the SCF complex as well as IκB (Figure 7D and Figure S3C) even upon infection.

These results indicated that SteA does not inhibit the binding of SCF complex to IκB, and SteA itself binds to the IκB-SCF complex.

### SteA Binds to Cullin-1

**Figure 8:**
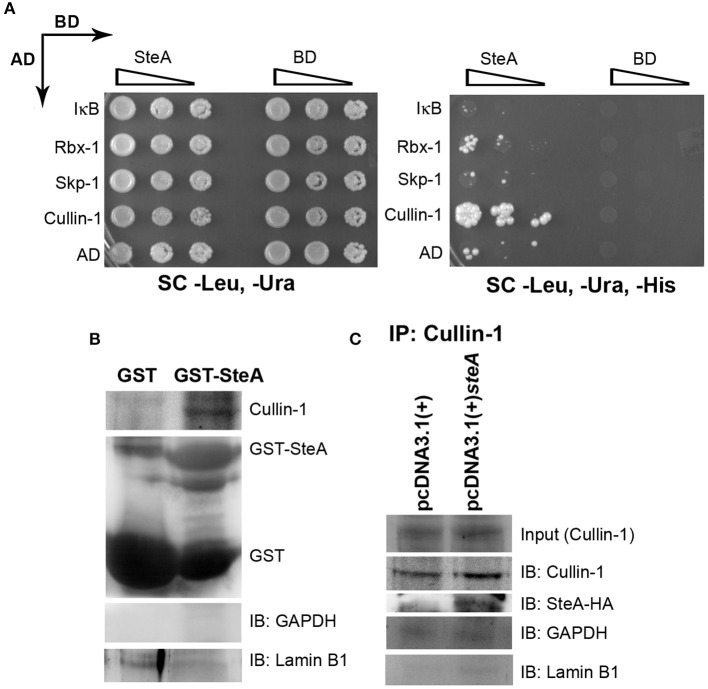
SteA binds to Cullin-1. **(A)** SteA interacts with Cullin-1. The members of the Skp-1, Cullin-1, F-box (SCF) complex and *steA* were cloned in vectors containing activation domain (AD) and binding domain (BD), respectively, for the yeast two-hybrid screen. These were then cotransformed in yeast and selected on synthetic complete (SC)-Leu, SC-Ura, and SC-Leu, SC-Ura, SC-His plates and incubated for 2 days at 30°C. The growth of yeast expressing Cullin-1 and SteA on SC-Leu, SC-Ura, and SC-His plates indicated interaction between SteA and Cullin-1. **(B)** Cullin-1 immunoprecipitates with SteA in the absence of stimulation. Whole-cell lysates of unstimulated RAW 264.7 cells were incubated with glutathione beads labeled with either GST-SteA or GST alone. The beads were then washed, and the proteins bound to GST-SteA or GST were analyzed by Western blotting using anti-Cullin-1 and anti-GST antibodies. **(C)** SteA interacts with Cullin-1. HEK 293 cells were transfected with pcDNA3.1(+)*steA* or pcDNA3.1(+) empty plasmid. After 36 h of transfection, whole-cell lysates were prepared following 30 min of tumor necrosis factor alpha (TNFα) stimulation and immunoprecipitated with anti-Cullin-1 antibody. HA-tagged SteA was detected using anti-HA antibody. **(B,C)** Glyceraldehyde 3-phosphate dehydrogenase (GAPDH) and Lamin B1 were also probed after IP to check the specificity of the pulldowns.

To better understand how SteA suppresses IκB degradation, we wanted to explore with which component of the SCF complex SteA binds or whether it directly binds to IκB. Toward this, we did a yeast two-hybrid screen for the interaction of SteA with IκB and the members of the SCF complex (Figure 8A). In this screen, we observed that SteA binds to Cullin-1 but not to IκB, Rbx-1, or Skp-1, indicating that SteA directly interacts with Cullin-1.

Interestingly, we also observed colocalization of SteA with Cullin-1 in both TNFα-stimulated and unstimulated SteA-transfected HEK 293 cells using confocal microscopy, suggesting an association of SteA with Cullin-1 even when SCF complex is not assembled at IκB (Figure S5A). Furthermore, we did a GST pulldown assay in whole-cell lysates of unstimulated RAW 264.7 cells using GST-tagged SteA and observed that Cullin-1 was coimmunoprecipitated with GST-tagged SteA (Figure 8B and Figure S4B). In addition, in HEK 293 cells expressing HA-tagged SteA, we observed that SteA coimmunoprecipitated with Cullin-1 upon immunoprecipitation with anti-Cullin-1 antibody (Figure 8C and Figure S4A).

Altogether, these data confirmed that SteA interacts with Cullin-1 to suppress IκB degradation.

### SteA Binds to Cullin-1 and Prevents Its Neddylation by Interfering With the Dissociation of Cand-1 From Cullin-1

**Figure 9:**
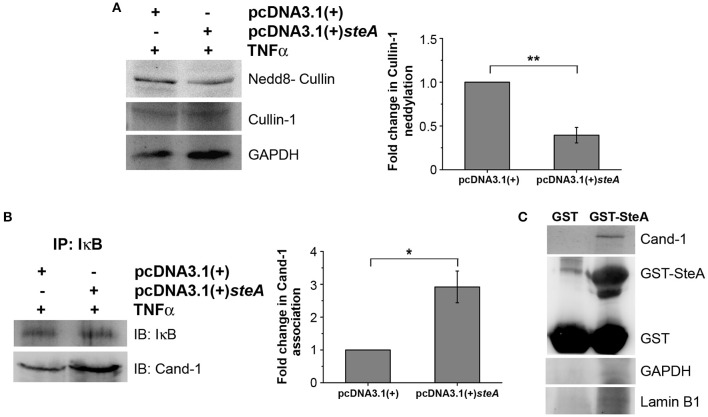
SteA suppresses the neddylation of Cullin-1 and the dissociation of Cand-1. **(A)** Neddylation of Cullin is suppressed in mammalian cells endogenously expressing SteA. HEK 293 cells were transfected with pcDNA3.1(+)*steA* or pcDNA3.1(+) empty plasmid. After 24 h of transfection, whole-cell lysates were prepared following 30 min of tumor necrosis factor alpha (TNFα) stimulation and analyzed by Western blotting using anti-nedd8-Cullin and anti-Cullin-1 antibodies and densitometry. Glyceraldehyde 3-phosphate dehydrogenase (GAPDH) was used as a loading control. **(B)** Cand-1 dissociation from SCF-E3 complex localized at IκB is decreased in the presence of SteA. HEK 293 cells were transfected with pcDNA3.1(+)*steA* or pcDNA3.1(+) empty plasmid. After 24 h of transfection, whole-cell lysates were prepared following 20 min of TNFα stimulation in the presence of proteasomal inhibitor (MG132) and immunoprecipitated with anti-IκB antibody. Cand-1 association was analyzed by Western blotting and densitometry. **(A,B)** For densitometric analysis, fold change was calculated with respect to empty plasmid transfected cells. Bar graphs represent mean ± SEM from three independent experiments. *p* values were calculated using Student's *t* test [*p < 0.05, **p < 0.01, ***p < 0.001, ns *p* > 0.05 vs. pcDNA3.1(+) transfected cells]. **(C)** Cand-1 coimmunoprecipitates with SteA even in the unstimulated cells. Whole-cell lysates of unstimulated RAW 264.7 cells were incubated with glutathione beads labeled with either GST-SteA or GST alone. The beads were then washed, and the proteins bound to GST-SteA or GST were analyzed by Western blotting using anti-Cand-1 and anti-GST antibodies. GAPDH and Lamin B1 were also probed after IP to check the specificity of the pulldown.

Generally, upon assembly of the SCF complex to IκB, Cullin-1 gets neddylated. This neddylation of Cullin-1 is a crucial step for the activation of the SCF-E3 ligase, which leads to the ubiquitination of IκB (39). Upon neddylation, Cand-1, which is bound to Cullin-1 in the inactivated state, is removed from the E3 ligase complex (40). Therefore, we first checked the neddylation of Cullin-1 in HEK 293 cells transfected with pcDNA3.1(+) and pcDNA3.1(+)*steA* and observed that, upon TNFα stimulation, Cullin-1 was less neddylated in cells expressing SteA (Figure 9A). Furthermore, to check if Cand-1 dissociation from the E3 ligase complex was inhibited in the presence of SteA, we stimulated SteA-expressing HEK 293 cells after pretreatment with MG132 and immunoprecipitated with anti-IκB antibody and probed for Cand-1. We observed that Cand-1 dissociation was lesser in the presence of SteA (Figure 9B). In addition, in GST pulldown assay, we observed the association of Cand-1 to GST-tagged SteA even in unstimulated RAW 264.7 cells, indicating that SteA remains associated with Cullin-1/Cand-1 complex even without stimulation (Figure 9C and Figure S4C). To further confirm this, we checked the colocalization of SteA and Cand-1 in SteA-transfected HEK 293 cells, and we observed no difference in the colocalization of SteA and Cand-1 with or without TNFα stimulation (Figure S5B).

**Figure 10:**
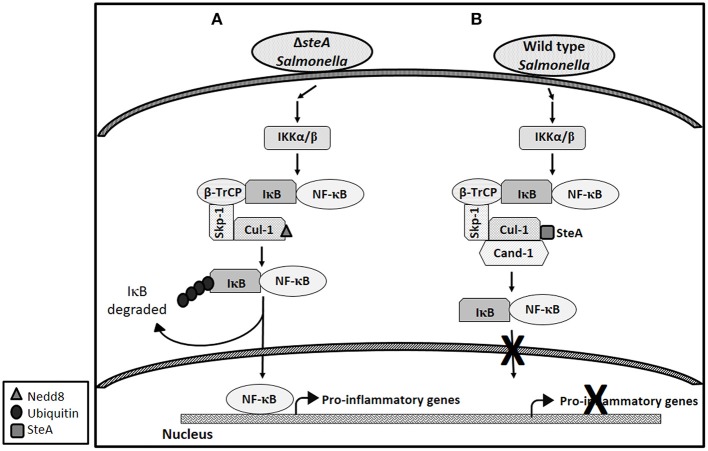
Schematic representation of how SteA suppresses the nuclear factor kappa B (NF-κB) pathway. **(A)** In absence of SteA, *Salmonella* Typhimurium (Δ*steA*) infection triggers a signaling cascade, which leads to the phosphorylation of IKKα/β. IKKα/β then phosphorylates IκB, which is the inhibitor of NF-κB. In the inactivated state, IκB is bound to NF-κB, thus preventing NF-κB to translocate to the nucleus. Phosphorylated IκB is recognized by the F-box protein β-TrCP, which further leads to recruitment of the Skp-1 and Cullin-1 (Cul-1) to form the SCF-E3 ligase complex. Cullin-1 is bound to Cand-1 in the inactive state, and upon neddylation, Cand-1 dissociates from Cullin-1 leading to the complete activation of the E3 ligase. The E3 ligase complex is responsible for the polyubiquitination of IκB. Upon polyubiquitination, IκB undergoes proteasomal degradation, rendering NF-κB free to translocate to the nucleus and transcribe proinflammatory genes. **(B)** During wild-type (wt) *Salmonella* Typhimurium infection (when SteA is present), SteA binds to Cullin-1 (of SCF-E3 ligase) and suppresses the neddylation of Cullin-1 and the dissociation of Cand-1 from Cullin-1, thereby suppressing the polyubiquitination of IκB. This leads to the suppression of NF-κB-mediated proinflammatory responses.

Altogether, this study shows that SteA suppresses the immune responses of the host by suppressing the degradation of IκB. SteA prevents the degradation of IκB by binding to Cullin-1 and thus suppressing the neddylation of Cullin-1, which is necessary for the complete activation of the E3 ligase complex to ubiquitinate IκB (Figure 10).

## Discussion

In response to a bacterial infection, the immune cells of the host elicit a proinflammatory response and signal other cells of the intrusion. This eventually leads to clearing of the bacteria from the host system. Some pathogens including *Salmonella* Typhimurium have been known to evade the immune system of the host and establish a niche in the macrophages. *Salmonella* Typhimurium makes use of some of the effectors that it translocates directly into the host cells via the type three secretion systems (T3SS) to suppress the host's immune responses. The effectors secreted by T3SSs, mainly T3SS-1, play different roles in immunomodulation of the host (8). Toward characterizing the T3SS-1 role of SteA, we found that mice infected with *steA*-deficient *Salmonella* Typhimurium (Δ*steA*) showed lower survival as compared to the wt *Salmonella* Typhimurium infected mice (Figures 1A,B). Furthermore, a higher immune response in mice infected with Δ*steA* seemed to be the cause of increased lethality (Figures 1C–E and Figure S1). Similarly, higher proinflammatory responses were observed when RAW 264.7 murine macrophage cell line and BMDMs were infected with Δ*steA* as compared to wt-infected cells (Figure 2A). Furthermore, we confirmed that SteA-mediated suppression of immune response is a T3SS-1 effect using a strain deficient in T3SS-2 (Δ*spi2*) and a Δ*spi2*Δ*steA* double mutant (Figure 2B).

The proinflammatory responses are mediated by a multistep signaling cascade involving the activation of the NF-κB pathway or the MAP kinase pathway leading to the activation of transcription factors NF-κB and AP-1 (36). Some of the effectors of *Salmonella* Typhimurium are already known to suppress the host-immune responses. For example, SpvC suppresses the MAP kinase pathway by dephosphorylating JNK and ERK (41), SptP acts on the ERK activation (42, 43), while AvrA suppresses both the NF-κB and the MAP kinase pathway (44–47), hence suppressing inflammation. To be able to understand how SteA suppresses the proinflammatory responses, we first checked the activation levels of p38 and JNK MAP kinases in the whole-cell lysates and the levels of NF-κB family members (p65 and c-Rel) in the nuclear lysates. We observed that SteA does not affect the MAP-kinase activation but acts on the NF-κB pathway to suppress the immune responses (Figures 3–5). To confirm our observation, we transfected HEK 293 and RAW 264.7 cells with NF-κB luciferase reporter plasmid and found the activation of NF-κB to be higher upon infection with Δ*steA* than the wt (Figure 5B). Furthermore, upon endogenous expression of SteA in HEK 293 cells, the NF-κB reporter activity was found to be suppressed (Figure 5C). Since suppression of the NF-κB pathway is crucial for the survival of *Salmonella* Typhimurium in the host, *Salmonella* Typhimurium secretes various effectors to control it at multiple levels. In the inactive state, NF-κB remains bound to the inhibitor IκB; for the activation of NF-κB, IκB kinase (IKK) phosphorylates IκB and leads to its ubiquitination and degradation rendering NF-κB free. The free NF-κB transcription factor translocates to the nucleus, binds to the DNA, and upregulates transcription of inflammatory cytokine genes (37). Some effectors such as AvrA and SseL are known to interfere with IκB degradation by deubiquitinating IκB (44, 48). Another effector, SpvD, affects the recycling of NF-κB, and PipA acts as a protease, which cleaves RelA, a subunit of NF-κB after its activation (49, 50). Toward exploring how SteA suppresses the NF-κB pathway, we checked the degradation level of IκB in the presence or absence of SteA and observed increased degradation of IκB in the absence of SteA compared to when SteA was present, suggesting that SteA acts on IκB degradation (Figure 4). The degradation of IκB is a multistep pathway which is initiated by the phosphorylation of IκB. IκB phosphorylation is dependent on IKK; therefore, there could be increased activation of IKK itself in the absence of SteA, but we observed comparable activation of IKK in cells infected with wt or Δ*steA* strains (Figure 6A). Following phosphorylation, the SCF-E3 ligase complex assembles on IκB, leading to the polyubiquitination of IκB and hence its proteasomal degradation (31). One of the effectors, GogB, suppresses ubiquitination by acting on Skp-1, a component of SCF-E3 ligase (51). Our study indicated that SteA also binds to the SCF complex and suppresses the activation of IκB by interfering with the ubiquitination (Figure 6B). However, we observed that SteA mainly acts on Cullin-1 of SCF-E3 ligase complex (Figure 8). For ubiquitination to happen, the neddylation of Cullin-1 follows the assembly of SCF-E3 ligase complex to IκB. Neddylation of Cullin-1 is a crucial step for the complete activation of the E3 ligase (39). Cullin-1 in the inactive form remains bound to Cand-1; however, upon activation, Cand-1 dissociates from Cullin-1 allowing neddylation to happen (40). Our study indicated that SteA binds to Cullin-1 (Figure 8), thus interfering with neddylation of Cullin-1 and the dissociation of Cand-1 from Cullin-1 (Figure 9). This further results in suppression of IκB ubiquitination and its degradation.

In response to *Salmonella* Typhimurium infection, proinflammatory responses are generated in the host. This immune response is capable of clearing the infection; also, if unchecked, it may be lethal for the host. However, for *Salmonella* Typhimurium to be able to survive and multiply in the host, these proinflammatory responses need to be suppressed. For this, *Salmonella* Typhimurium secretes an array of effectors which suppress the immune responses at multiple levels and ensures its survival in the host.

Altogether, our study shows that *Salmonella* Typhimurium effector SteA suppresses the proinflammatory responses generated against *Salmonella* Typhimurium by suppressing the neddylation of Cullin-1 and hence suppressing the degradation of IκB, eventually leading to the suppression of NF-κB activation (Figure 10).

## Data Availability Statement

All datasets generated for this study are included in the article/Supplementary Material. The raw datasets will be available on request.

## Ethics Statement

The animal study was reviewed and approved by the Institutional Animals Ethics Committee (IAEC) of the Indian Institute of Science Education and Research, Mohali.

## Author Contributions

AG designed and performed experiments, analyzed the data, and wrote the paper. RS performed experiments and analyzed the data. AM designed experiments, analyzed data, supervised the study, and wrote the paper.

### Conflict of Interest

The authors declare that the research was conducted in the absence of any commercial or financial relationships that could be construed as a potential conflict of interest.

## Abbreviations

Δ*steA*: deletion mutant of steA gene
compl: *steA* complemented in the Δ*steA* background
compl-H: 6X His-tagged *steA* complemented in the Δ*steA* background.
