# Supplementary material for: Salmonella Effector SteA Suppresses Proinflammatory Responses of the Host by Interfering With IκB Degradation
Source: Front Immunol. 2019 Dec 10;10:2822. doi: 10.3389/fimmu.2019.02822 (PMC6914705; doi:10.3389/fimmu.2019.02822)
Supplement: Supplementary file 1 [file Data_Sheet_1.PDF]

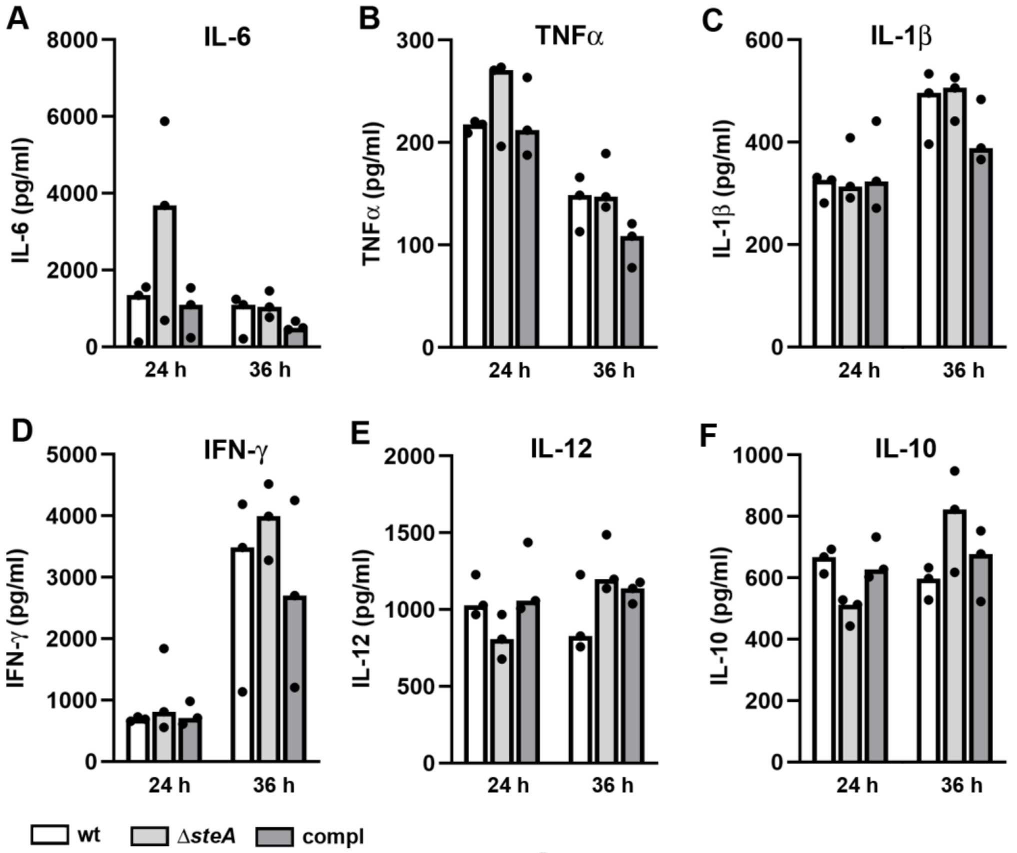

Figure S1

Figure S1. An increase in IL-6 (A), TNFα (B) at 24 h and IFN-γ (D), IL-12 (E) and IL-10 (F) at 36 h were observed in the serum of mice infected with Δ*steA* compared to the wt- or compl-infected mice. (A-F) 6-8 weeks old Balb/c mice were infected with (5 X 10^5) wt, Δ*steA* or compl. Blood was collected at 24 h.p.i and 36 h.p.i. Serum was analysed for various cytokines using ELISA. Bar graphs represent median of three mice (each mouse represented as •) in each group.

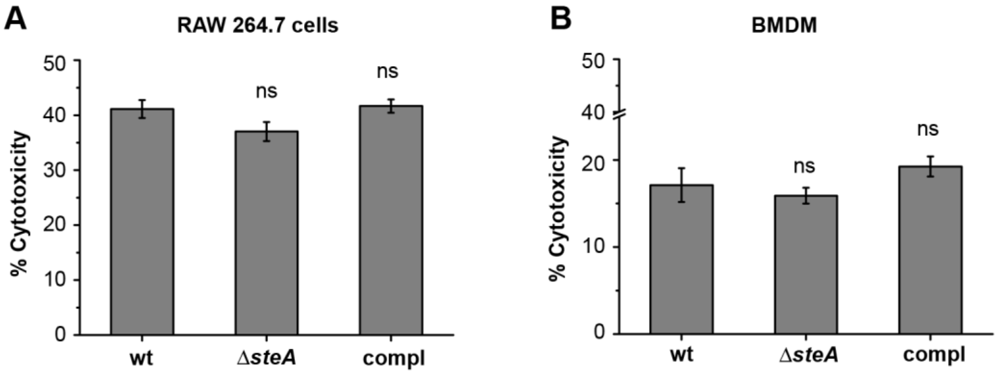

Figure S2

Figure S2. No significant difference in cell cytotoxicity of wt-, Δ*steA*- or compl-infected RAW 264.7 cells (A) or BMDMs (B). (A-B) RAW 264.7 cells or BMDMs were infected with wt, Δ*steA* or compl at an MOI of 20:1 or 10:1 respectively. After 8 h, the cell cytotoxicity was measured using LDH release assay. Bar graphs represent mean ± SEM from three independent experiments. P values were calculated using one-way ANOVA (*p<0.05, **p< 0.01, ***p<0.001, ns p>0.05 versus wt-infected cells).

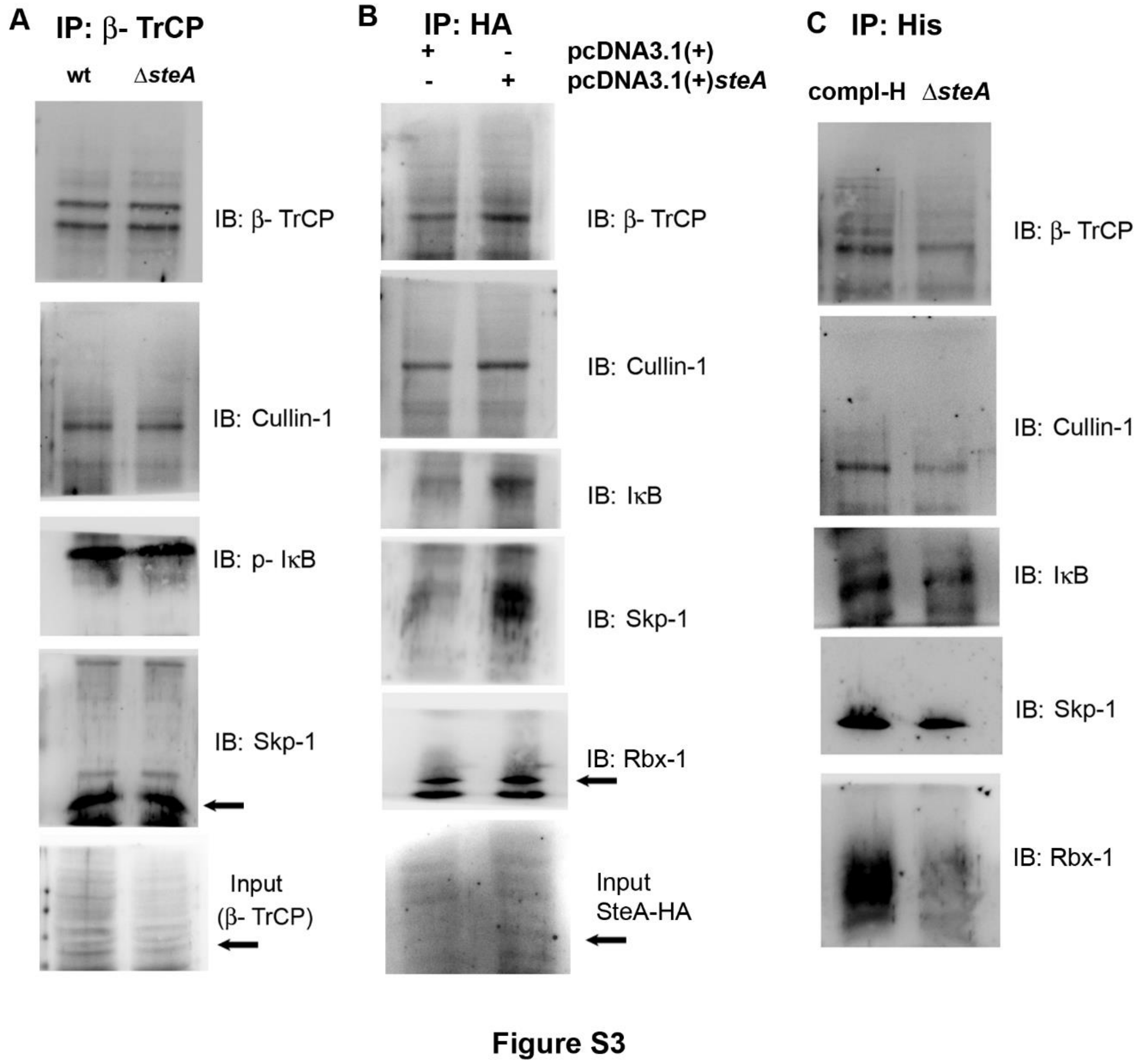

Figure S3. Full blots for immunoprecipitation using anti-βTrCP (A), anti-HA (B) and anti-His (C) antibodies. (A-C) After immunoprecipitation, the lysates were run on the SDS-PAGE gel and transferred to a PVDF membrane. Then the blot was cut (depending on the expected molecular weights) before incubation with antibody and then probed for different co-immunoprecipitated proteins, such as, β-TrCP, Cullin-1, IκB, Skp-1 or Rbx-1.

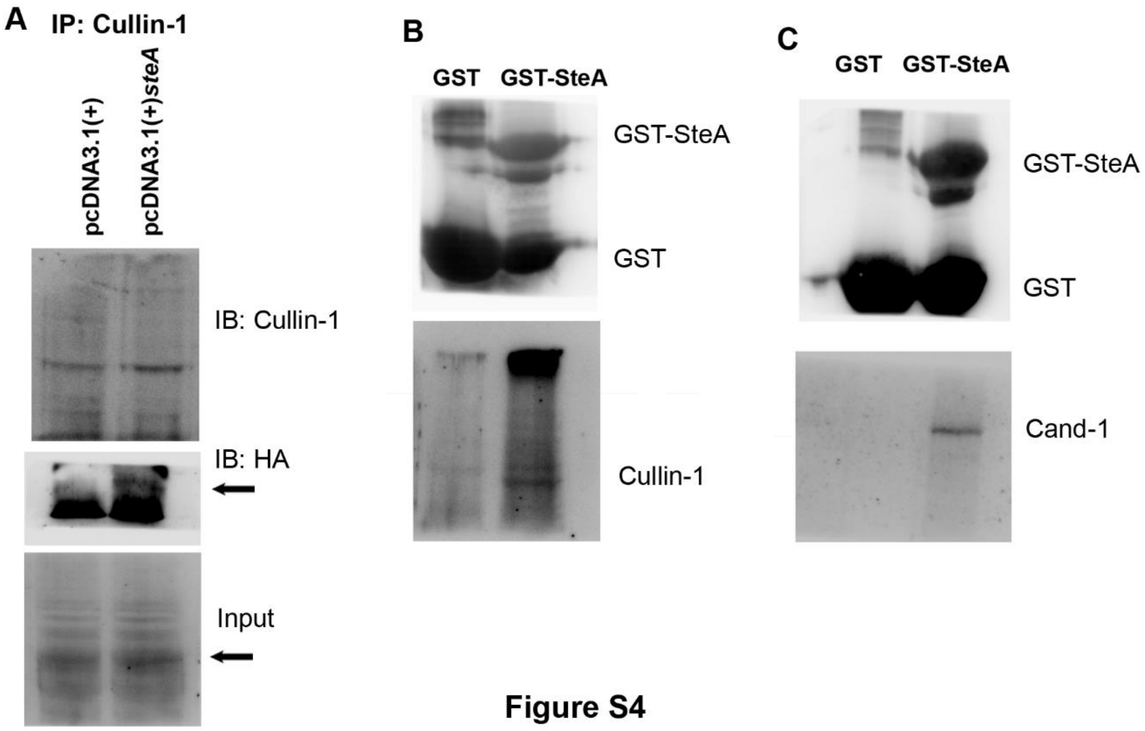

Figure S4. (A) Full blots for immunoprecipitation using anti-Cullin-1 antibody. After immunoprecipitation, the lysates were run on the SDS-PAGE gel and transferred to a PVDF membrane. The blot was then cut (depending on the expected molecular weights) and probed for Cullin-1and SteA-HA (using anti-HA antibody). (B-C) Full blots for GST pull-down with GST and GST-SteA. After GST pull-down, the lysates were run on the SDS-PAGE gel and transferred to a PVDF membrane. The blot was then cut (depending on the expected molecular weights) and probed for anti-GST, anti-Cullin-1 (B) or anti-Cand-1 (C).

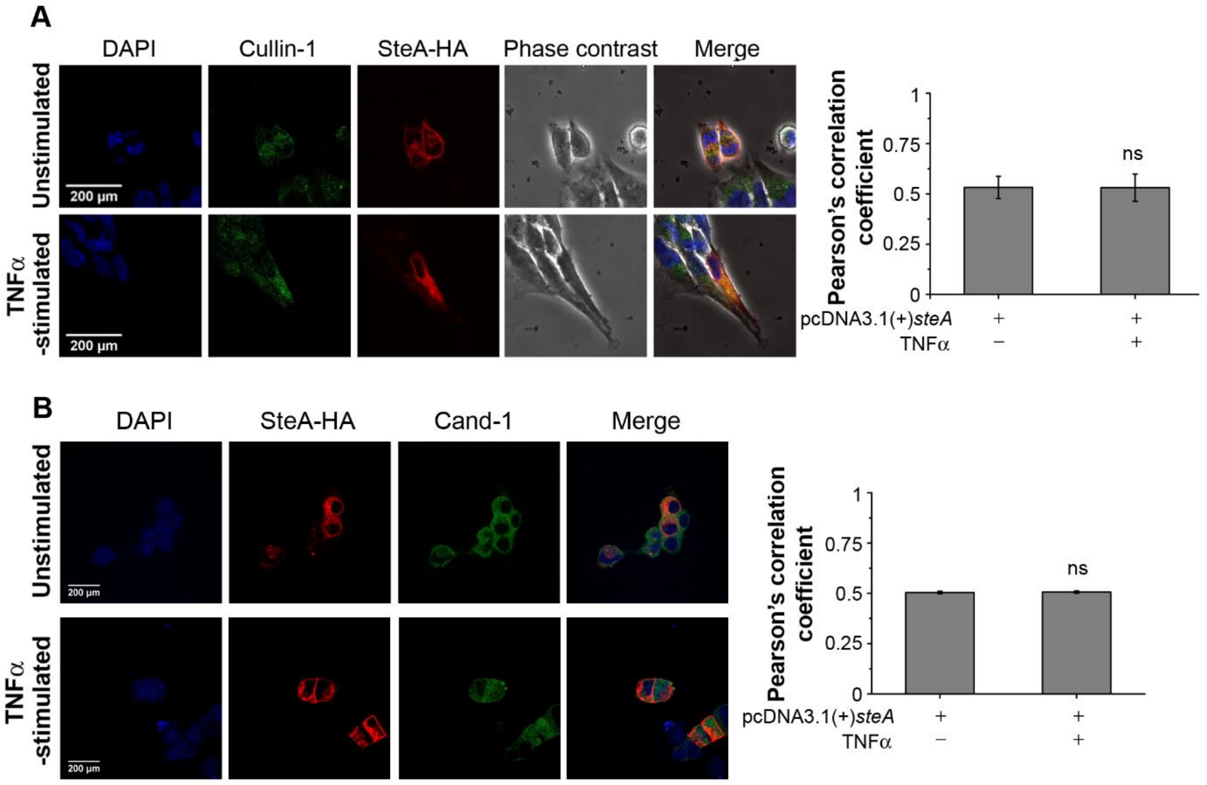

Figure S5

Figure S5. SteA co-localizes with Cullin-1(A) and Cand-1(B) irrespective of TNFα stimulation. (A-B) HEK 293 cells overexpressing HA-tagged SteA were fixed after 30 min of TNFα stimulation and were incubated with anti-Cullin-1(A) or anti-Cand-1(B) and anti-HA primary antibodies. Then, the cells were stained with Alexa 488-tagged (for Cullin-1 or Cand-1), Alexa 568-tagged (for HA) secondary antibodies and DAPI (for the nucleus). The cells were then observed under a confocal microscope. The co-localization was quantified using Pearson's correlation coefficient (PCC) taking 8-10 fields per experiment. Bar graphs represent mean ± SEM from three independent experiments. P values are calculated using Student's t-test (ns $p>0.05$ versus PCC in unstimulated cells).
